# Application of LogitBoost Classifier for Traceability Using SNP Chip Data

**DOI:** 10.1371/journal.pone.0139685

**Published:** 2015-10-05

**Authors:** Kwondo Kim, Minseok Seo, Hyunsung Kang, Seoae Cho, Heebal Kim, Kang-Seok Seo

**Affiliations:** 1 Department of Agricultural Biotechnology and Research Institute for Agriculture and Life Sciences, Seoul National University, Seoul 151–921, Republic of Korea; 2 C&K Genomics Inc., 514 Main Bldg., Seoul National University Research Park, San 4–2 Bongcheon-dong, Gwanak-gu, Seoul 151–919, Republic of Korea; 3 Interdisciplinary Program in Bioinformatics, Seoul National University, Seoul 151–747, Republic of Korea; 4 Department of Animal Science and Technology, College of Life Science and Natural Resources, Sunchon National University, Suncheon, 540–742, Republic of Korea; CSIRO, AUSTRALIA

## Abstract

Consumer attention to food safety has increased rapidly due to animal-related diseases; therefore, it is important to identify their places of origin (POO) for safety purposes. However, only a few studies have addressed this issue and focused on machine learning-based approaches. In the present study, classification analyses were performed using a customized SNP chip for POO prediction. To accomplish this, 4,122 pigs originating from 104 farms were genotyped using the SNP chip. Several factors were considered to establish the best prediction model based on these data. We also assessed the applicability of the suggested model using a kinship coefficient-filtering approach. Our results showed that the LogitBoost-based prediction model outperformed other classifiers in terms of classification performance under most conditions. Specifically, a greater level of accuracy was observed when a higher kinship-based cutoff was employed. These results demonstrated the applicability of a machine learning-based approach using SNP chip data for practical traceability.

## Introduction

Due to the occurrence of animal-related diseases such as bovine spongiform encephalopathy (BSE) and avian influenza (AI), consumer attention to food quality has increased greatly. Accordingly, place of origin (POO) tracing systems have become important to increasing consumer confidence regarding food safety. In the food industry, these are referred to as traceability systems. Traceability is defined as a method that can guarantee the identification of animals or animal products within the food industry [1]. This system is already mandatory for most animal products in a large number of countries. Product tracking has conventionally been conducted by labeling with ear tags and tattoos [1, 2]. Although this technique presents several advantages, including easy application, low cost, and fast data processing, it is vulnerable to fraud or loss [1]. Thus, genetic traceability has been proposed as an alternative to conventional traceability systems. Genetic traceability is the same as labeling systems in principle, except that DNA is used to identify animals or their products. It is possible to distinguish individual animals from one another based on DNA [3]. Moreover, DNA molecules are difficult to falsify, can withstand various processes within the food distribution system, and can be extracted from different types of tissues [1, 4]. These advantages have led to increased application and research into use of DNA markers for traceability. One typical marker, the single nucleotide polymorphism (SNP), has been widely applied [5–7]. There are several methods for obtaining SNP information regarding a sample, including next generation sequencing (NGS), microarrays, and SNP chips. Among these, genotyping using a SNP chip is less expensive and produces SNP data for a relatively large number of samples by customizing chip design.

Numerous studies have been conducted to develop prediction models for classification using diverse biomarkers [8–10]; however, few of these have focused on traceability. One reason for this is that traceability involves multiclass classification. Multiclass classification is generally associated with several difficulties [11]. The main problem associated with this type of classification is optimization. For example, when training sets are given, minimization of the loss function should be performed to build an accurate classifier. Loss function is affected by the number of classes, and minimization of this obstacle could be attained by reducing the number of classes. Several classifiers such as the K-nearest neighbor (KNN) and support vector machine (SVM) have frequently been employed to overcome problems related to multiclass classification [12–14]. Some studies have used KNN and SVM to classify foods according to origin [15, 16]. In addition, LogitBoost can address multiclass classification problems using a parametric method [17, 18].

In the present study, we genotyped 4,122 pigs that originated from 104 farms using a customized SNP chip. Based on these data, we attempted to develop a POO prediction model considering three variable factors: (1) Kinship-based filtering was applied to assess the applicability of classification-based approaches for practical POO prediction; (2) the wrapper-method was used as a feature selection step to remove redundant features [19]; (3) LogitBoost, SVM, and KNN were used as classifiers. We compared classification performance using combinations of these factors to identify the optimal POO prediction model.

## Materials and Methods

### Prescreening SNP markers to generate the customized SNP chip

A total of 384 pigs belonging to five major commercial breeds (19 Korean native black pigs, 17 Landrace, 168 Yorkshire, 84 Berkshire, and 96 Duroc) were genotyped using an Illumina Porcine SNP60 chip to prescreen SNP markers. SNPs were filtered according to several criteria (minor allele frequency [MAF] ≤ 0.05, missing rate ≥ 0.10, and Hardy-Weinberg equilibrium test p-value ≤ 0.001). Following this filtering step, we retrieved 39,785 SNPs for Korean native black pigs, 42,156 SNPs for Landrace, 44,961 SNPs for Yorkshire, 41,408 SNPs for Berkshire, and 39,652 SNPs for Duroc. Among these, 312 SNP markers that were identified in five breeds (MAF ≥ 0.4) were retrieved, and four to nine SNPs with lower linkage disequilibrium (LD) were selected for each chromosome. As a result, 133 SNP markers were obtained. We next performed additional genotyping for 1,045 muscle tissue samples obtained from 11 slaughterhouses (detailed information regarding slaughterhouses is provided in S1 Table) throughout the Republic of Korea to confirm that the selected SNP markers were evenly distributed for each location. Ultimately, 96 SNP markers including known SNPs for individual animal identification were selected while taking into account the geographical distribution of SNP markers (0.3 ≤ allele frequency ≤ 0.7). These 96 SNP markers were used as features in downstream analyses, including feature selection and classification. More detailed information regarding these markers is presented in S2 Table. All genotyped samples were obtained from pigs slaughtered for meat production.

### Genotyping 96 SNPs and kinship coefficient-based subset generation for development of the traceability prediction model

From April to June 2014, 4,122 slaughtered commercial pigs originating from 104 different farms were genotyped using a customized SNP chip manufactured by Illumina (provided by the S1 Dataset). Some individual animals and SNPs were filtered out (MAF < 0.01 and genotype missing rate > 0.9) using PLINK v1.07 [20]. As a result, 3,974 individual pigs and 92 SNPs remained.

Most pigs in the livestock industry are derived from a crossbred population, and sires and semen are shared with several farms. Therefore, the origins of pigs are not clearly distinguishable because of genetic similarity. However, sows are generally not shared among farms and produce piglets several times during their lives [21, 22]. Therefore, we assumed that piglets produced from a single sow might have genetically close relationships. In practice, because genetic information regarding sows in a farm could be considerably dissimilar, it is necessary to screen farms consisting of unrelated individuals to distinguish pigs according to their farms. We employed kinship coefficients [23] to evaluate the genetic relationships. The King 1.4 software was used to calculate pairwise kinship coefficients within each farm [23]. The relationship between two individuals is classified by a kinship coefficient > 0.353 as monozygotic twins (0.177, 0.353), as parent-offspring or sibling pairs (0.088, 0.177), as second-degree relative pairs (such as half-siblings, avuncular pairs or grandparent-grandchild pairs; 0.044, 0.088) or as third-degree relative pairs (such as first cousins), while < 0.044 indicates unrelated pairs [23].

To infer the degree of genetic relatedness to attain reasonable classification accuracy, we generated four subsets of data composed of farms to satisfy the following criteria: mean of the kinship within a farm ≥ 0.00, 0.05, 0.10, and 0.15. The subset with a kinship mean ≥ 0.00 had 741 individuals from twenty farms, the subset with a kinship mean ≥ 0.05 included 235 individuals from eight farms, the subset with a kinship mean ≥ 0.10 included 134 individuals from five farms, and the subset with a kinship mean ≥ 0.15 contained 67 individuals from two farms. To visualize the distribution of individuals by their genetic information in the four subsets, scatter plots were generated by principal component analysis (PCA) using A Tool for Genome-wide Complex Traits Analysis (GCTA) [24].

### Wrapper-based feature selection for removing redundant SNP markers

Feature selection is an important step for improving classification performance. Although we already performed a prescreening step to generate an SNP marker set suitable for traceability, redundant or irrelevant features might be included in this set. Therefore, we utilized the wrapper method [25] to extract valuable features. The wrapper method is a classifier-dependent approach designed to search for feature subsets that would produce the best accuracy. There were two approaches for extracting the best feature subset. The first was top-down selection for which a model was evaluated after eliminating one feature from the entire feature set and replacing the eliminated feature with another. This process was repeated for all features (Approach 1). The second approach was bottom-up selection in which the evaluation step was conducted using only one feature (Approach 2).

### Classifiers for multiclass prediction

One main reason for the limited research on traceability prediction is that this type of prediction presents a representative multiclass classification problem. For multiclass data, classification is often associated with several difficulties [26, 27]. Unfortunately, most traditional classifiers were developed for binary classification, which cannot be directly employed for multiclass prediction. There are two approaches for addressing multiclass classification problems. The first is a one-against-all approach employing binary classifiers such as the support vector machine (SVM) and LogitBoost [28]. The second is use of classifiers able to predict multiclass data, such as the k-nearest-neighbor (KNN).

LogitBoost is a recently developed boosting algorithm that can handle multiclass problems by considering multiclass logistic loss [17, 18]. This technique has been used to predict protein structural classes known as representative multiclass problems [29]. Other approaches, including SVM- and KNN-based multiclass prediction, have been implemented in many fields [12–14]. KNN, which is one of the simplest methods, classifies an instance according to a majority vote of its k nearest instances. SVM is a high-performance classifier that builds an optimal hyperplane containing the largest distances from support vectors in each given class. As a result, spaces distinguished based on the hyperplanes represent specific classes and predict unknown class data (test data).

In the present investigation, we used these classifiers with the following parameters: LogitBoost *I* = 20, KNN (IBk) *k* = 11, and SVM (SMO) *kernel* = Radial Basic Function (RBF) Kernel, which is implemented in the RWeka [30] package of the R software. These parameter values were determined based on the results of a greedy search using various parameter values for each classifier (S2 Fig). The default for the RWeka package was used for all other parameters.

### Comparison of classification performance

We compared the classification performance of three classifiers (LogiBoost, KNN, and SVM) according to classification accuracy [31], balanced accuracy [32], sensitivity [31], specificity [31], area under the curve (AUC) values [33], and a receiver operating characteristic (ROC) curve [31] with 10-fold cross-validation to avoid overfitting. ROC curves were generated by calculating the false positive and true positive rates for continuous thresholds. We used the *ROCR* package [34] of the R software to calculate and visualize the ROC curves.

### Simulation analysis for estimating the effects of biases

To investigate the effects of biases generated by the various sample sizes and number of classes in different kinship-based subsets, we performed a simulation analysis. We used the LogitBoost classifier and 92 features to estimate biases in the simulation analysis. Three types of simulations were carried out. Whole simulations were repeated 1000 times using sampling without replacement, and 10-fold cross-validations were performed to analyze classification accuracies for each repetition. The first simulation was conducted to survey the impact of the number of classes. To assess this effect, we adjusted the number of classes in the whole kinship-based subsets to two, which was the smallest value among subsets. We then randomly selected two classes for each repetition. Sample sizes varied according to random sampling. The second simulation was conducted to survey the effects of sample size. We fixed the sample size at 67, which was the smallest value among all of the subsets. The numbers of classes varied according to random sampling. Finally, we simultaneously investigated the effects of two biases by adjusting both sample size and number of classes.

## Results and Discussion

### Assessment of prediction model performance for traceability classification

**Fig 1:**
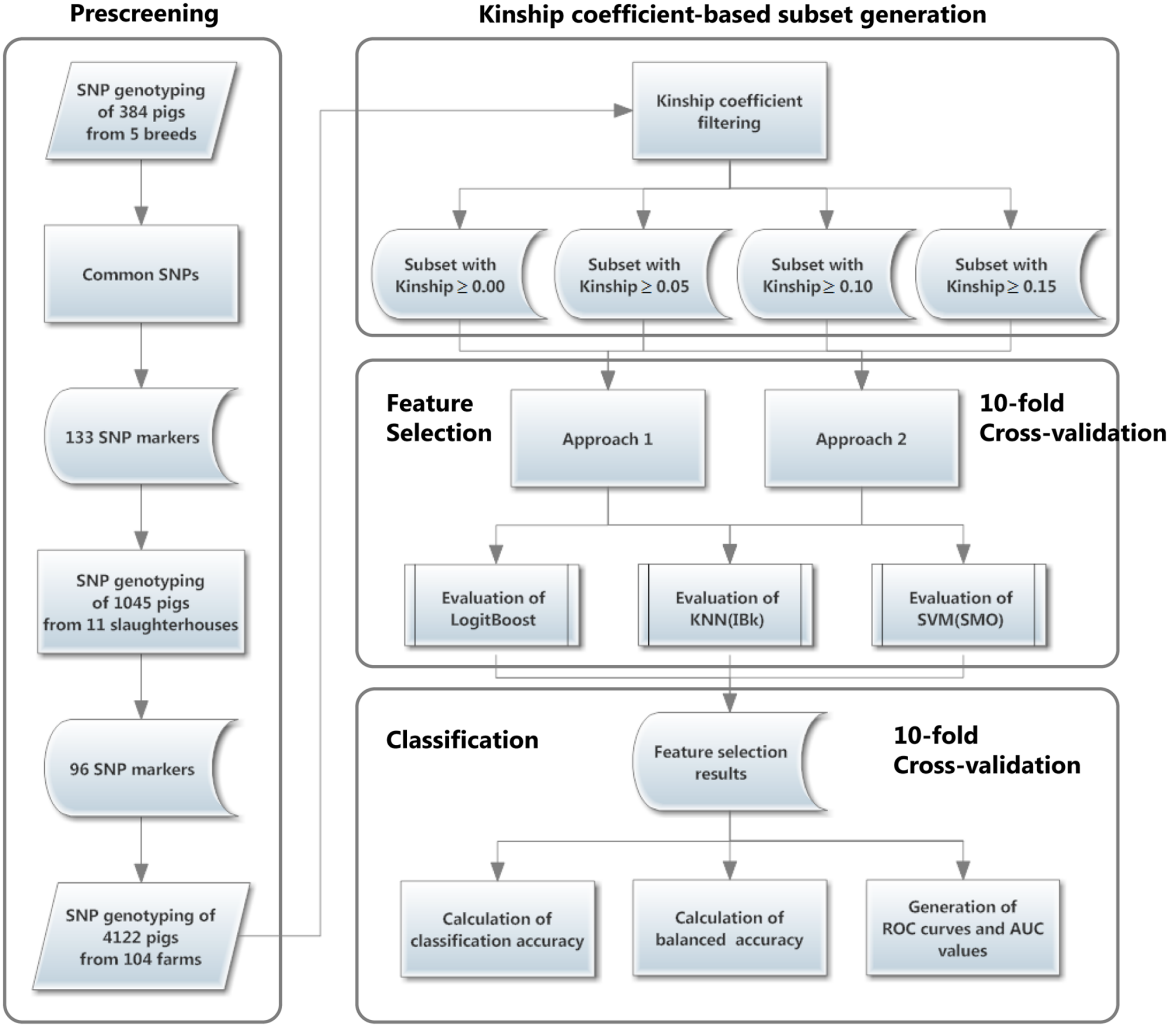
A diagram representing the processes of building the prediction model for traceability. The prescreening process for selecting the SNP markers consists of two major steps: retrieval of common SNPs for five pig breeds and selection of SNP markers based on geographical distribution (farm location). Farms were filtered by the kinship coefficient mean and four subsets were generated. The feature selection process for removing redundant features was performed using two approaches (detailed descriptions of these techniques are provided in the manuscript) and three classifiers. Using the selected features, classification performance was evaluated based on three factors (classification accuracy, balanced accuracy, and ROC curves).

**Fig 2:**
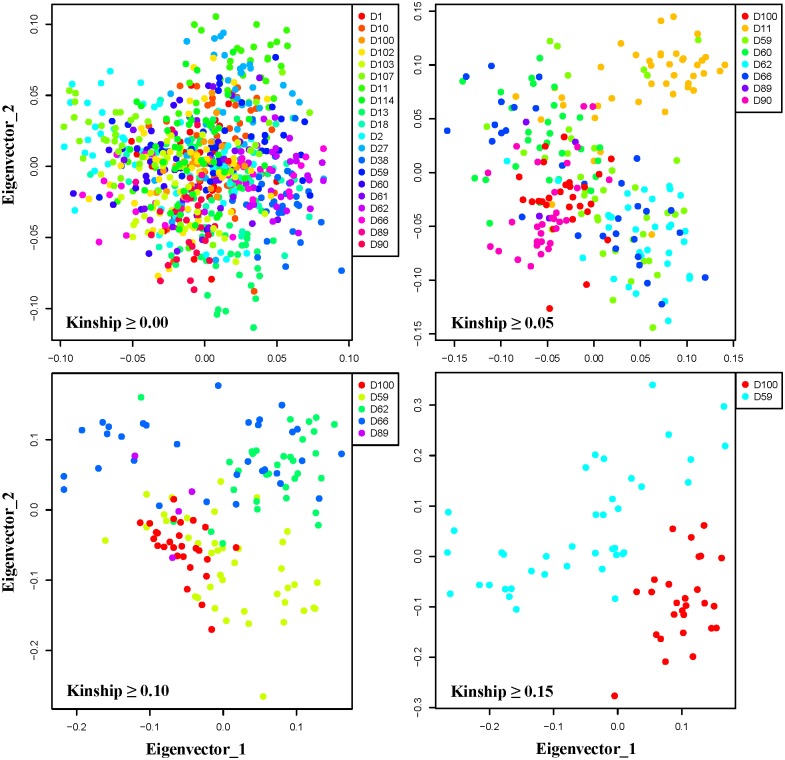
Scatter plots for four subsets with different kinship coefficient criteria (X-axis: Eigen vector 1 and Y-axis: Eigen vector 2). Scatter plots were generated by PCA using GCTA [24]. Each point represents an individual animal and is colored based on the farm information. When the kinship cutoff increased, each farm was more clearly distinguishable.

In the present study, we applied three representative multiclass classifiers to four subsets of SNP data based on kinship-based filtering. In addition, 2 (top-down and bottom-up) × 3 (LogitBoost, SVM, and KNN) wrapper-based feature selection methods were used to generate the best prediction model for traceability. The entire pipeline for data processing including classification is presented as a schematic diagram in Fig 1. Specific elements (classifier, feature subset, and kinship coefficient) were expected to be directly associated with prediction accuracy. We investigated the influence of these elements by calculating the prediction accuracy from various points of view. First, we determined how distinguished the individual animals were according to the farms of origin using four subsets based on kinship coefficient-based filtering. As shown in Fig 2, the four subsets established based on the cutoff criteria (mean of the kinship within a farm ≥ 0.00, 0.05, 0.10, and 0.15, respectively) were visualized by PCA. As the cutoff criterion increased, greater segregation among farms was observed. These findings imply that traceability prediction could be performed when individuals on one farm have highly similar genetic information, which was expected. Using the PCA, we observed subsets with different numbers of samples and farms depending on the cutoff criterion. Therefore, these figures should be interpreted with caution in terms of bias due to the smaller number of classes, larger sample size, and larger number of features, which generally improve accuracy when classification is performed.

We next calculated the power of explanation for traceability prediction for each SNP. We defined “feature score” as the contribution of a feature to the accuracy for classification. In Approach 1, a feature score was calculated based on the accuracy of whole features minus the accuracy associated with eliminating a feature. For Approach 2, a feature score was the accuracy associated with using that feature. As expected, only a few outliers were observed for all feature scores (S3 Table and S1 Fig). Most outliers fell below the lower quantile, indicating that the majority of prescreened features were well selected (S1 Fig). If the prescreening step had not identified meaningful features for POO prediction, outliers would be observed below the lower quantile and above the upper quantile due to randomness. Therefore, we confirmed that the customized chip containing 96 SNPs was suitable for POO prediction. We also demonstrated that some features should be removed from the prediction model for better accuracy.

**Fig 3:**
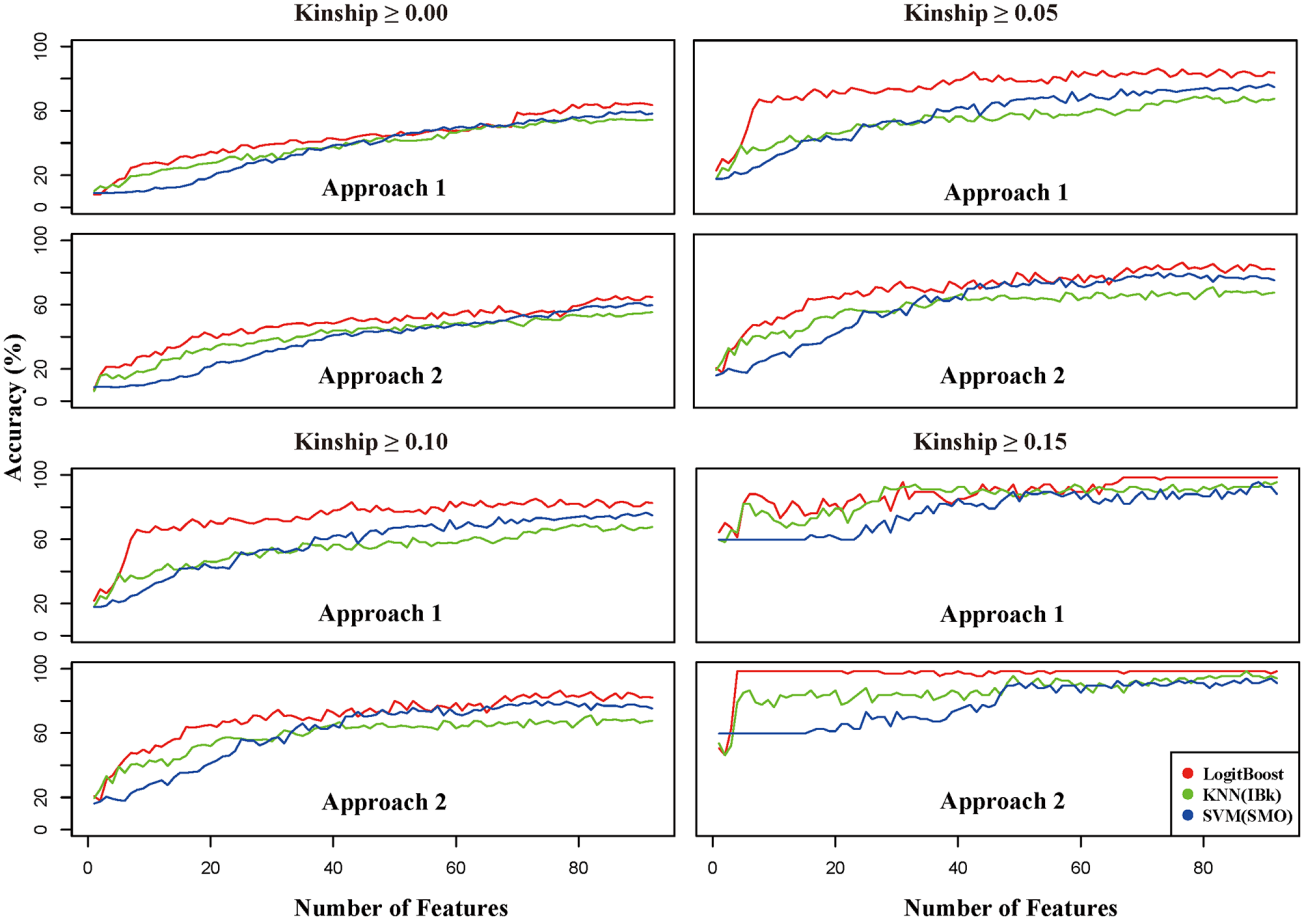
Line plots for comparing classification accuracy according to several factors, including classifiers, feature subsets, and kinship-based filtered subsets. The X-axis contains the number of features (1 to 92 SNPs), while the Y-axis shows classification accuracy. Approach 1 is the top-down feature selection method while Approach 2 is the bottom-up feature selection technique. LogitBoost-based classification accuracy is represented by the red line. Lines corresponding to the KNN and SVM classification methods are green and blue, respectively.

We next performed feature selection before carrying out classification analysis. As shown in Fig 3, we calculated the classification accuracy for the different classifiers and the number of features (features were added to the feature set for the prediction model in order of the feature score generated in Approaches 1 and 2). Four subsets were used to compare classification performance depending on the classifiers and feature sets. Accuracy was determined using 10-fold cross-validation to avoid overfitting. As expected, a subset with more features and higher kinship had better classification accuracy. Overall, we observed a pattern in which accuracy gradually increased with the number of features. These findings indicated that the customized chip was appropriately designed for traceability because only a few irrelevant features might be included for the 96 SNPs. Generally, including a large number of irrelevant features in a whole feature-set does not increase accuracy, although the features are included in the prediction model. Thus, we again concluded that the 96 pre-selected SNPs were suitable for traceability.

Interestingly, the LogitBoost classifier showed better performance in terms of accuracy than the other classifiers in most situations. This remarkable result indicated that the LogitBoost classifier was more suitable for predicting animal or food origin. It is difficult to constantly obtain a better performance with a specific classifier in diverse situations, as shown by comparison of the SVM and KNN classifiers. Nevertheless, with the exception of one situation (kinship ≥ 0.15 and Approach 1), the LogitBoost classifier consistently performed better than the others. In addition, the classification accuracy achieved with LogitBoost had a smaller variance than that of the other classifiers in most situations (Table 1). LogitBoost also outperformed the other classifiers in terms of efficiency, with greater levels of accuracy observed when using a relatively small number of features. Overall, the results of this study demonstrated that LogitBoost appears to be the best method for POO prediction in terms of performance assessment when using accuracy as a measurement.

**Table 1:** Best classification accuracies for diverse situations (two different feature selection approaches, four different kinship filtered sets, and three classifiers). Levels of accuracy were calculated by 10-fold cross-validation and expressed as the means ± 10-fold variance. Bold represents greater accuracy than other classifiers for each kinship-based filtered subset.

		Approach 1		Approach 2	
Kinship	Algorithm	# of Features	Mean ± Variance	# of Features	Mean ± Variance
	LogitBoost	**83**	**0.652 ± 0.002**	**81**	**0.661 ± 0.004**
≥ 0.00	KNN (IBk)	80	0.557 ± 0.006	90	0.549 ± 0.001
	SVM (SMO)	86	0.588 ± 0.004	87	0.578 ± 0.001
	LogitBoost	**72**	**0.878 ± 0.002**	**85**	**0.868 ± 0.005**
≥ 0.05	KNN (IBk)	88	0.720 ± 0.015	81	0.726 ± 0.010
	SVM (SMO)	88	0.784 ± 0.004	90	0.747 ± 0.009
	LogitBoost	**47**	**0.950 ± 0.002**	**64**	**0.942 ± 0.003**
≥ 0.10	KNN (IBk)	24	0.833 ± 0.013	28	0.850 ± 0.005
	SVM (SMO)	73	0.792 ± 0.009	50	0.790 ± 0.008
	LogitBoost	**53**	**0.992 ± 0.001**	**4**	**0.992 ± 0.001**
≥ 0.15	KNN (IBk)	82	0.983 ± 0.003	20	0.992 ± 0.001
	SVM (SMO)	73	0.967 ± 0.005	72	0.909 ± 0.007

Although classification accuracy is good for evaluating classifiers, unintended bias is occasionally generated. For example, intact accuracy may produce misleading information about general performance when a classifier is evaluated using an imbalanced dataset. In such cases, classification accuracy is not a reliable measure for assessing a prediction model. To avoid an inflated performance estimation for imbalanced data, we employed another measure, balanced accuracy. The balanced accuracies were calculated as the average accuracies for each class. To further compare classification accuracy and balanced accuracy, we calculated balanced accuracies for the same combinations of factors as shown in Table 1. As indicated in Table 2, LogitBoost still generally produced better results than the other methods in terms of balanced accuracy. The D89 class that contained only four individual animals always showed poor performance, as indicated by a balanced accuracy of zero. Although the D89 class had a high kinship coefficients mean, PCA demonstrated that individuals in this class overlapped entirely with individuals in other classes (Fig 2). Collectively, the characteristics of the D89 class including small sample size and an overlap of individuals with animals from other classes caused poor performance in terms of balanced accuracy. We also found that the D62 class had particularly high balanced accuracy for the LogitBoost classifier throughout all kinship-based subsets. For example, the LogitBoost classifier had a balanced accuracy of 0.923 for the kinship-based subset (kinship ≥ 0.00), while the KNN- and SVM-based approaches had balanced accuracy values of 0.469 and 0.475, respectively. This phenomenon was consistently observed for the other kinship-based subsets.

**Table 2:** Evaluation of predicted performance according to balanced accuracy. The balanced accuracies were calculated by 10-fold cross-validation. Values represent the mean ± 10-fold variance. Figures written in bold represent a higher level of balanced accuracy than those of the other classifiers in each class. Figures given in parentheses represent the number of features used in classifiers.

Kinship ≥ 0.00						
	Approach 1			Approach 2		
Class	LogitBoost (83)	KNN (IBk) (80)	SVM (SMO) (86)	LogitBoost (81)	KNN (IBk) (90)	SVM (SMO) (87)
D1	**0.387 ± 0.124**	0.050 ± 0.025	0.000 ± 0.000	**0.446 ± 0.146**	0.133 ± 0.104	0.017 ± 0.003
D2	0.683 ± 0.057	0.729 ± 0.039	**0.810 ± 0.035**	**0.829 ± 0.026**	0.808 ± 0.044	0.742 ± 0.030
D10	0.422 ± 0.068	0.421 ± 0.088	**0.701 ± 0.039**	0.513 ± 0.061	0.389 ± 0.114	**0.612 ± 0.032**
D11	**0.713 ± 0.033**	0.676 ± 0.107	0.695 ± 0.042	**0.735 ± 0.036**	0.624 ± 0.100	0.625 ± 0.108
D13	0.751 ± 0.039	0.823 ± 0.024	**0.905 ± 0.011**	0.703 ± 0.025	0.810 ± 0.027	**0.913 ± 0.018**
D18	**0.418 ± 0.041**	0.245 ± 0.071	0.277 ± 0.117	**0.547 ± 0.048**	0.254 ± 0.032	0.291 ± 0.109
D27	**0.540 ± 0.067**	0.532 ± 0.065	0.513 ± 0.076	**0.585 ± 0.083**	0.416 ± 0.051	0.521 ± 0.062
D38	**0.774 ± 0.045**	0.712 ± 0.074	0.682 ± 0.064	**0.857 ± 0.057**	0.685 ± 0.051	0.697 ± 0.074
D59	**0.797 ± 0.060**	0.642 ± 0.069	0.648 ± 0.098	**0.768 ± 0.050**	0.698 ± 0.047	0.677 ± 0.063
D60	**0.755 ± 0.091**	0.067 ± 0.044	0.145 ± 0.038	**0.611 ± 0.114**	0.108 ± 0.034	0.361 ± 0.118
D61	**0.605 ± 0.150**	0.462 ± 0.160	0.150 ± 0.114	0.185 ± 0.082	**0.273 ± 0.044**	0.000 ± 0.000
D62	**0.923 ± 0.017**	0.469 ± 0.064	0.475 ± 0.131	**0.840 ± 0.040**	0.608 ± 0.062	0.486 ± 0.066
D66	**0.655 ± 0.082**	0.448 ± 0.170	0.340 ± 0.062	**0.463 ± 0.115**	0.407 ± 0.038	0.411 ± 0.145
D89	0.000 ± 0.000	0.000 ± 0.000	0.000 ± 0.000	0.000 ± 0.000	0.000 ± 0.000	0.000 ± 0.000
D90	0.693 ± 0.080	**0.827 ± 0.038**	0.527 ± 0.132	0.708 ± 0.104	**0.717 ± 0.068**	0.689 ± 0.133
D100	0.638 ± 0.082	**0.889 ± 0.035**	0.663 ± 0.094	0.705 ± 0.110	**0.833 ± 0.125**	0.746 ± 0.048
D102	**0.770 ± 0.037**	0.355 ± 0.055	0.683 ± 0.120	**0.862 ± 0.030**	0.364 ± 0.038	0.702 ± 0.049
D103	0.452 ± 0.031	**0.607 ± 0.095**	0.492 ± 0.045	0.418 ± 0.100	**0.517 ± 0.081**	0.468 ± 0.100
D107	0.733 ± 0.063	**0.787 ± 0.045**	0.757 ± 0.040	**0.818 ± 0.076**	0.718 ± 0.080	0.795 ± 0.024
D114	**0.766 ± 0.056**	0.630 ± 0.077	0.678 ± 0.050	0.683 ± 0.102	0.628 ± 0.055	**0.747 ± 0.040**
Balanced Accuracy	**0.624 ± 0.061**	0.518 ± 0.067	0.507 ± 0.065	**0.614 ± 0.070**	0.500 ± 0.060	0.525 ± 0.061
**Kinship ≥ 0.05**						
	**Approach 1**			**Approach 2**		
Class	LogitBoost (72)	KNN (IBk) (88)	SVM (SMO) (88)	LogitBoost (85)	KNN (IBk) (81)	SVM (SMO) (90)
D11	**0.975 ± 0.006**	0.940 ± 0.018	0.933 ± 0.028	0.975 ± 0.006	**0.980 ± 0.004**	0.933 ± 0.012
D59	0.793 ± 0.103	**0.904 ± 0.029**	0.832 ± 0.032	**0.938 ± 0.010**	0.848 ± 0.032	0.751 ± 0.057
D60	**0.843 ± 0.029**	0.150 ± 0.065	0.597 ± 0.142	**0.717 ± 0.073**	0.204 ± 0.045	0.575 ± 0.132
D62	**0.955 ± 0.009**	0.767 ± 0.063	0.727 ± 0.052	**0.963 ± 0.006**	0.693 ± 0.066	0.718 ± 0.100
D66	**0.900 ± 0.024**	0.661 ± 0.061	0.696 ± 0.080	**0.795 ± 0.116**	0.591 ± 0.081	0.623 ± 0.090
D89	0.000 ± 0.000	0.000 ± 0.000	0.000 ± 0.000	0.000 ± 0.000	0.000 ± 0.000	0.000 ± 0.000
D90	0.838 ± 0.035	0.802 ± 0.076	**0.947 ± 0.014**	0.875 ± 0.045	0.806 ± 0.106	**0.944 ± 0.014**
D100	0.900 ± 0.044	**0.941 ± 0.015**	0.867 ± 0.048	**0.967 ± 0.011**	0.889 ± 0.111	0.817 ± 0.114
Balanced Accuracy	**0.776 ± 0.031**	0.646 ± 0.041	0.700 ± 0.049	**0.779 ± 0.033**	0.626 ± 0.056	0.670 ± 0.065
**Kinship ≥ 0.10**						
	**Approach 1**			**Approach 2**		
Class	LogitBoost (47)	KNN (IBk) (24)	SVM (SMO) (73)	LogitBoost (64)	KNN (IBk) (28)	SVM (SMO) (50)
D59	**1.000 ± 0.000**	0.896 ± 0.022	0.947 ± 0.007	**1.000 ± 0.000**	0.925 ± 0.016	0.950 ± 0.013
D62	**1.000 ± 0.000**	0.917 ± 0.031	0.745 ± 0.051	**1.000 ± 0.000**	0.942 ± 0.016	0.922 ± 0.017
D66	**0.942 ± 0.016**	0.785 ± 0.060	0.847 ± 0.046	**0.930 ± 0.027**	0.848 ± 0.028	0.677 ± 0.108
D89	0.000 ± 0.000	0.000 ± 0.000	0.000 ± 0.000	0.000 ± 0.000	0.000 ± 0.000	0.000 ± 0.000
D100	**0.950 ± 0.025**	0.933 ± 0.020	0.811 ± 0.050	**0.963 ± 0.012**	0.900 ± 0.026	0.622 ± 0.129
Balanced Accuracy	**0.778 ± 0.008**	0.706 ± 0.026	0.670 ± 0.031	**0.779 ± 0.008**	0.723 ± 0.017	0.634 ± 0.053
**Kinship ≥ 0.15**						
	**Approach 1**			**Approach 2**		
Class	LogitBoost (72)	KNN (IBk) (88)	SVM (SMO) (88)	LogitBoost (85)	KNN (IBk) (81)	SVM (SMO) (90)
D59	0.950 ± 0.025	**0.989 ± 0.001**	0.988 ± 0.002	**1.000 ± 0.000**	**1.000 ± 0.000**	0.975 ± 0.006
D100	0.852 ± 0.031	**0.933 ± 0.020**	0.858 ± 0.037	**0.950 ± 0.025**	0.875 ± 0.051	0.775 ± 0.068
Balanced Accuracy	0.901 ± 0.028	**0.961 ± 0.010**	0.923 ± 0.019	**0.975 ± 0.013**	0.938 ± 0.026	0.875 ± 0.037

LogitBoost-based approaches constantly showed better balanced accuracy than other techniques, except for the subset with a kinship mean ≥ 0.15, for which the KNN had a more balanced accuracy. However, the overall balanced accuracies were relatively low compared to analysis of the classification accuracy. These findings indicated that there were biases caused by imbalanced classes, which led to overestimation during analysis of the classification accuracy. Nevertheless, the findings from the accuracy and balanced accuracy analyses demonstrated that LogitBoost had better performance than the other methods, with a few exceptions. Overall, LogitBoost appears to be a more suitable model for POO prediction in terms of consistency. We also found that balanced accuracy increased with a higher mean kinship coefficient for the subsets.

**Fig 4:**
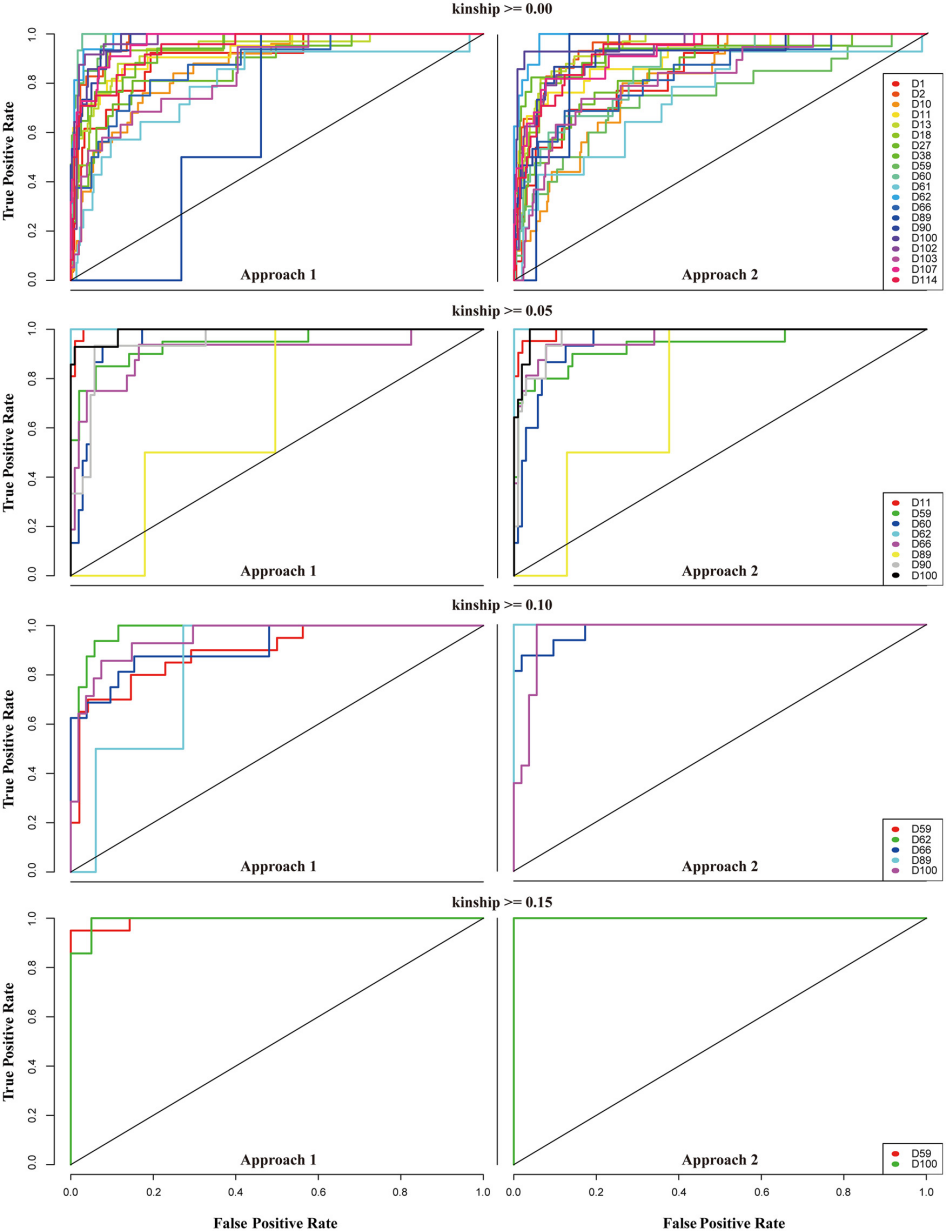
ROC curves for different kinship-based subsets to evaluate the suitability of specific farm groups with the LogitBoost classifier. To calculate sensitivity and specificity, data were divided in half and used as a training and test set. Threshold-specific performance could then be monitored using continuous cutoffs based on the ROC curves. All processes were conducted for the four subsets with two approaches. The D89 class showed the lowest performance in most cases.

By assessing the prediction model based on accuracy and balanced accuracy, we found that the LogitBoost classifier outperformed previously known classifiers for POO prediction. When balanced accuracy was used as a measurement, we also observed a strong class-specific accuracy pattern. To further investigate this pattern, ROC curves were produced as another technique for predicting performance (Fig 4). Strong farm-specific curves were observed. We again found that the D89 class had the lowest performance. The distinct difference between curves for the D89 class and those for the other classes can be interpreted as differences in suitability for the prediction model. Thus, ROC curves can be used to screen out a class that is unsuitable for the prediction model. Additionally, ROC curves showed better performance when the mean kinship coefficient increased, as indicated by the AUC values shown in S4 Table.

### Effects of biases of the kinship-based filtering approach on assessment of the prediction model

**Fig 5:**
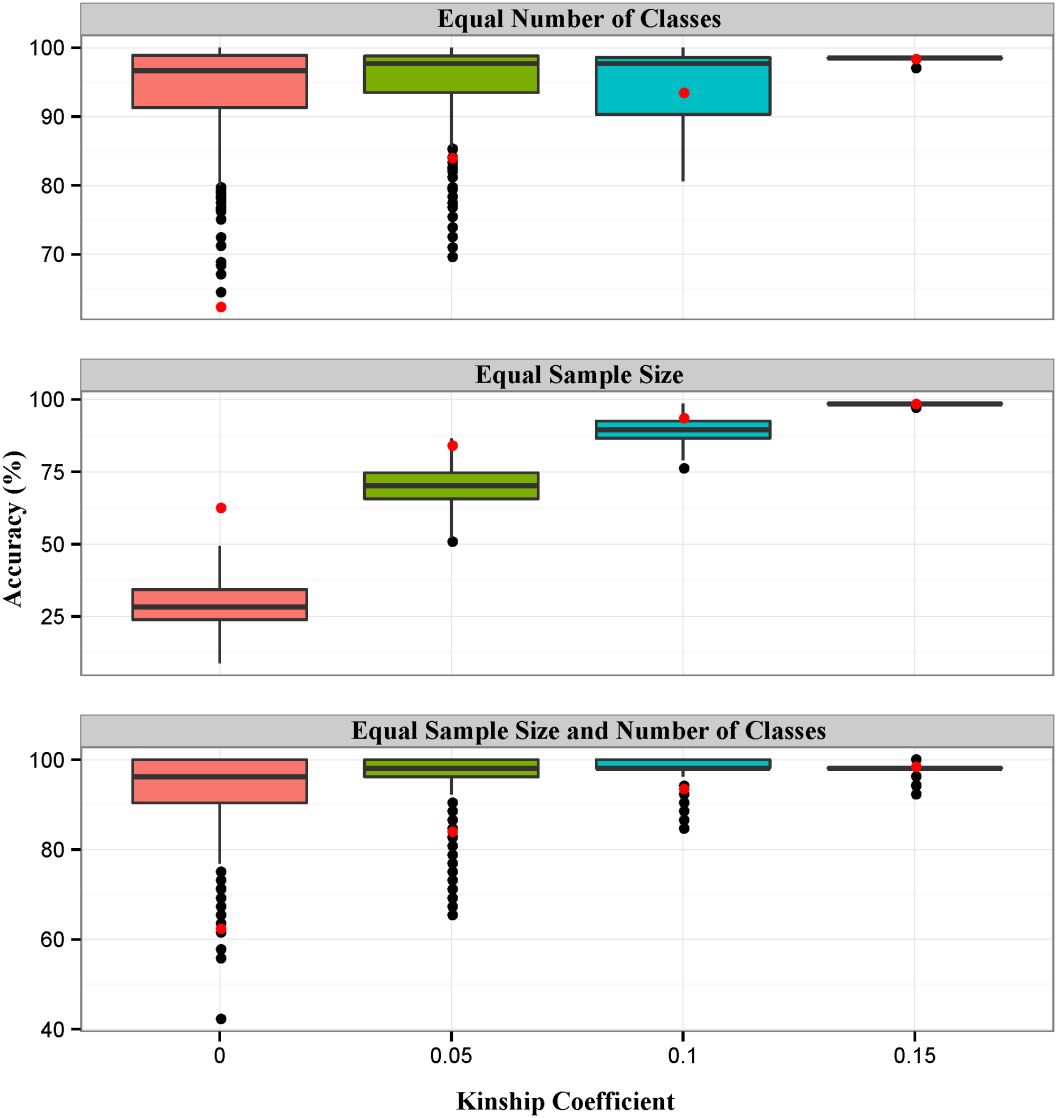
Results of sample size and number of classes correction. Data for the three simulation analyses were generated by adjusting three factors (sample size, number of classes, or both). For the top box-plot, sample size was set at 67, which was the smallest of the four subsets. For the middle box-plot, the number of classes was set at two, which was also the smallest for the four subsets. Finally, the bottom box-plot was generated using 26 samples (the smallest sample size among all classes) for each class (binary class). To determine the classification accuracies, 10-fold cross-validations were performed. All of these processes were conducted 1000 times using 92 features. Red dots represent the previously calculated observed accuracies.

Although several performance measures including accuracy, balanced accuracy, ROC curves, and AUC values showed better performance for POO prediction when the kinship cut-off criterion was greater, some bias-associated problems that prevented accurate model assessment remained. There are two types of bias, difference in sample size and difference in number of classes. In general, reducing the number of classes and/or a large training sample size leads to greater classification accuracy. In the current study, kinship-based filtering subsets had diverse sample sizes and numbers of classes. For this reason, our suggested kinship-based filtering approach was affected by the two types of bias, which represented a limitation of our study design. Therefore, we investigated the effects of the biases. To accomplish this, we performed three simulation analyses by adjusting the number of classes, the sample size, or both. The results of the first simulation analysis are shown in the top of Fig 5. The data in this figure confirmed that our previous assessment results were underestimated owing to the effects of the number of classes. The previous accuracies fell below median levels in all kinship subsets. In addition, four kinship-based subsets had similar median levels of accuracy when the number of classes was adjusted. Contrary to the first simulation, the second simulation showed that accuracies were overestimated because of the effects of sample size. As shown in Fig 5, the previous accuracies represented by red points were located above the median levels for all kinship subsets. In addition, the median accuracies for the four kinship-based subsets differed significantly. The two simulations described above confirmed that the number of classes has a significant influence on classification accuracy because accuracies in the second simulation varied more drastically according to differences in the number of classes, contrary to those in the first simulation. Finally, we simultaneously evaluated the effects of the two biases (as shown at the bottom of Fig 5), which revealed that the results were generally underestimated. However, the standard deviation of the accuracies decreased as the kinship coefficient cutoff increased.

Taken together, the results of the simulation studies indicated that the number of classes has a greater effect on classification accuracy than sample size. In addition, a higher kinship coefficient cutoff produced a lower standard deviation for the accuracies when both sample size and number of classes were constant. These findings indicated that we can expect to gain greater classification accuracy for populations with a higher kinship coefficient if the effects of sample size or number of classes are controlled. Although we controlled these biased factors in the simulation analysis, there was no practical method for fixing these two factors at equal values. This is because we did not collect samples while considering kinship coefficient values because the primary study design focused on identifying SNPs for individual identification. We actually screened the samples according to kinship coefficient after sample collection, which was a major limitation of our study. Nevertheless, the overall relationship between kinship coefficient and classification accuracy was consistent. Consequently, we determined that greater classification accuracy accompanied an increased kinship coefficient mean. We also obtained a reasonable accuracy distribution for the subset with a kinship coefficient greater than 0.10. These results imply that we can utilize a kinship coefficient of 0.10 as a criterion for pig traceability.

### Application of the prediction model for a practical traceability system

In this study, we concluded that the LogitBoost method was most suitable for POO prediction. LogitBoost has been utilized for various areas of data analysis such as protein structure prediction [29]. This method outperformed the SVM classifier for predicting protein structural classes. In addition, LogitBoost was employed for tumor classification using gene expression data [35]. Other types of data analysis such as text classification were also included in Logitboost applications [36]. Furthermore, the classifier has been employed in various fields that deal with multiclass prediction. Since POO prediction was also a representative type of multiclass classification, we anticipated that LogitBoost would be applicable. Not surprisingly, LogitBoost was successfully used for POO prediction. To the best of our knowledge, this is the first time the LogitBoost classifier has been implemented for traceability classification with genotyping data. Consequently, a few improvements should be made to enable the practical use of suggested approaches.

It is clear that when individual organisms originate from the same population they will have similar genotypes [37]. In the current study, we used kinship coefficients to measure the degree of the relationship between individuals based on this assumption. The results showed that subsets with a higher kinship coefficient had better performance. In particular, individuals within groups with a kinship coefficient higher than 0.1 were identified with reasonable accuracy using all of the evaluated statistics. If an original population was bound with an adequate relationship (pairwise kinship coefficient mean ≥ 0.10), it was possible to identify the original population of a given individual with reasonable accuracy. Our findings revealed that the suggested prediction model would be helpful for improving current traceability systems.

## Conclusion

In this study, we showed that the LogitBoost classifier had higher performance than other systems evaluated (KNN and SVM) using various performance measures and conditions. In addition, subsets with a higher kinship coefficient were shown to have better performance for POO prediction. These findings indicate that LogitBoost can be employed for traceability if an original population is genetically related. The findings of our study will provide a basis for improving existing traceability systems.

## Supporting Information

S1 Dataset Genotype data for 4,122 pigs originating from 104 farms. The first column contains individual identification data that includes farm information as “farm ID-individual ID”.
(XLSX)

S1 Fig Box-plots of feature scores calculated with three classifiers and two approaches. L, K, and S indicate LogitBoost, KNN, and SVM, respectively. 1 and 2 indicate Approach 1 and Approach 2, respectively.
(TIFF)

S2 Fig Line plots for the results of parameter optimization. The X-axis is the range of parameters used for each classifier (LogitBoost: iteration, KNN: K-nearest neighbors, and SVM: Kernel). The Y-axis represents classification accuracy calculated by 10-fold cross-validation.
(TIFF)

S1 Table Slaughterhouses. (DOCX)

S2 Table Selected SNP markers. Dashes indicate missing information.
(DOCX)

S3 Table Feature scores for each SNP calculated with three classifiers and two approaches. (DOCX)

S4 Table AUC values for each class calculated with LogitBoost and two approaches. (DOCX)
