# Supplementary figures and images for: Application of LogitBoost Classifier for Traceability Using SNP Chip Data
Source: PLoS One. 2015 Oct 5;10(10):e0139685. doi: 10.1371/journal.pone.0139685 (PMC4593556; doi:10.1371/journal.pone.0139685)

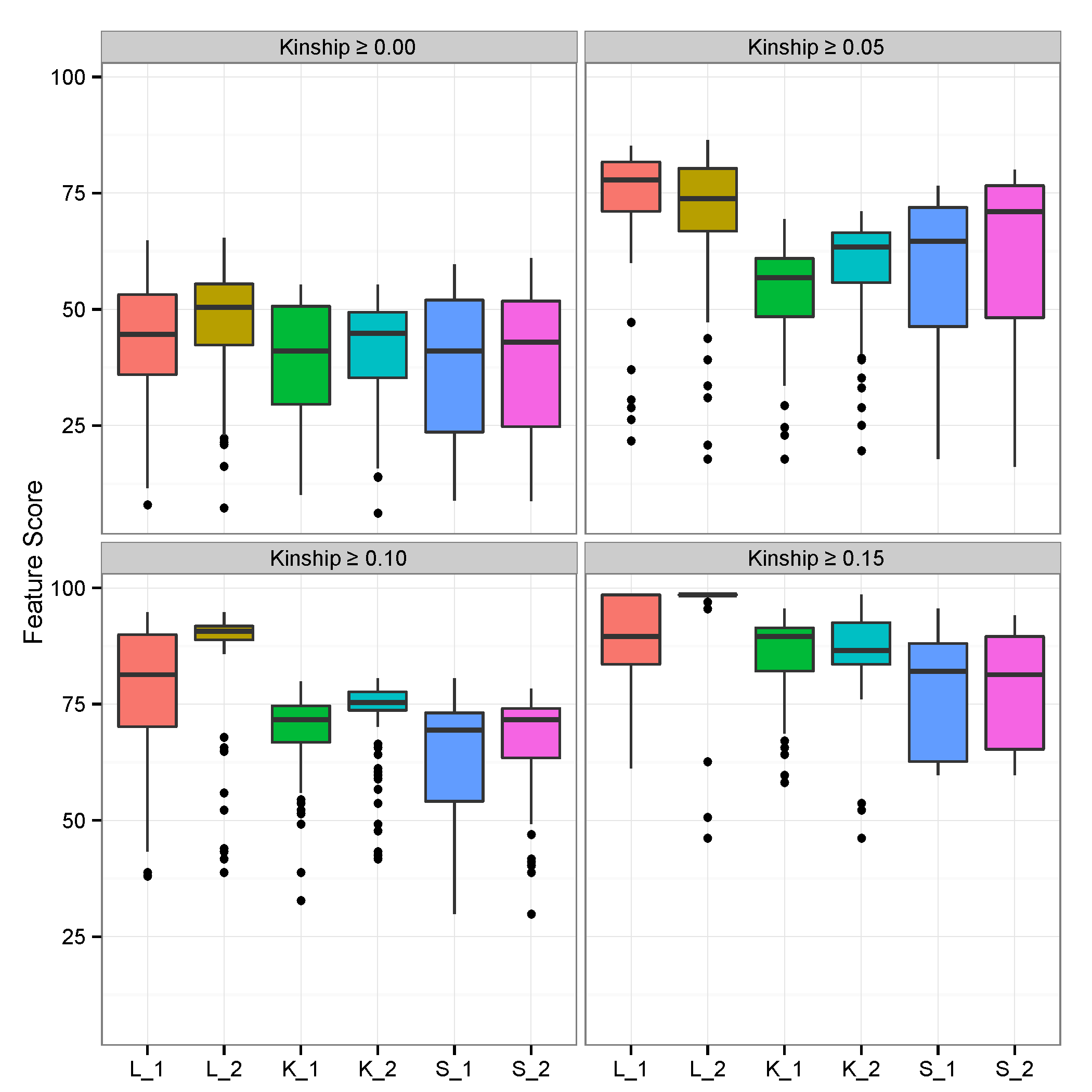

Supplement: S1 Fig — L, K, and S indicate LogitBoost, KNN, and SVM, respectively. 1 and 2 indicate Approach 1 and Approach 2, respectively. (TIFF) [file pone.0139685.s002.tiff]

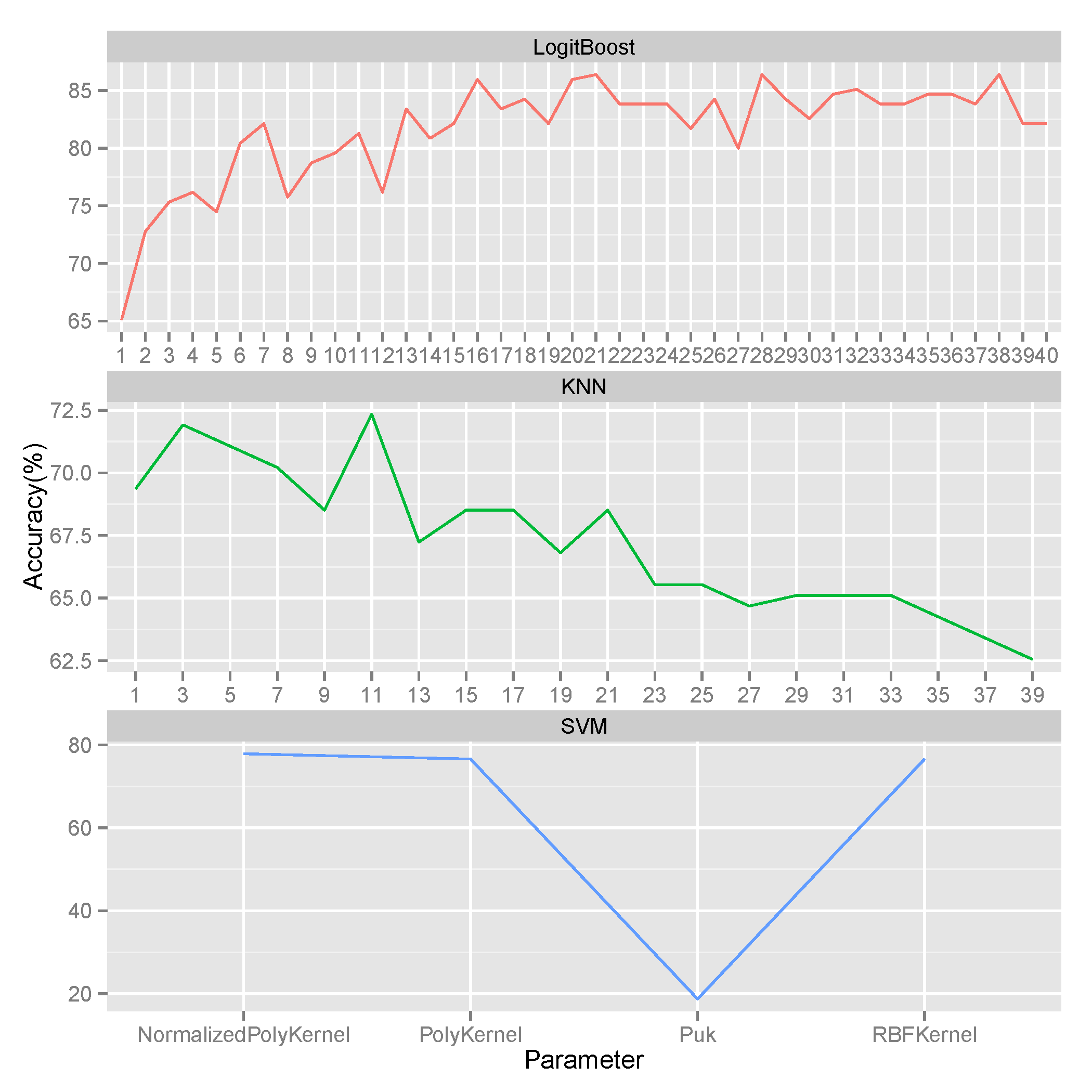

Supplement: S2 Fig — The X-axis is the range of parameters used for each classifier (LogitBoost: iteration, KNN: K-nearest neighbors, and SVM: Kernel). The Y-axis represents classification accuracy calculated by 10-fold cross-validation. (TIFF) [file pone.0139685.s003.tiff]
