# Supplementary material for: Application of LogitBoost Classifier for Traceability Using SNP Chip Data
Source: PLoS One. 2015 Oct 5;10(10):e0139685. doi: 10.1371/journal.pone.0139685 (PMC4593556; doi:10.1371/journal.pone.0139685)
Supplement: S1 Table — (DOCX) [file pone.0139685.s004.docx]

**S1 Table**. Slaughterhouses.

| **No.** | **Location** | **Slaughterhouse** | **Address** | **Phone** |
| --- | --- | --- | --- | --- |
| 1 | Gangwon-do | Gangwon LPC Inc. | 438-3, Gahyeon-dong, Wonju-si, Gangwon-do, Korea | 82-33-732-1300 |
| 2 | Gyeonggi-do | Bucheon Livestock Joint Market | 12-4, Samjeong-dong, Ojeong-gu, Bucheon-si, Gyeonggi-do, Korea | 82-32-620-5000 |
| 3 | Gyeonggi-do | Hyupsin Food Inc. | 298, Bakdal-ro, Manan-gu, Anyang-si, Gyeonggi-do, Korea | 82-31-447-9001 |
| 4 | Gyeongsangnam-do | Jinju SK Industry Inc. | 1369, Namgang-ro, Jinju-si, Gyeongsangnam-do, Korea | 82-55-755-5508 |
| 5 | Gyeongsangnam-do | Bukyung Livestock Joint Market | 6-9, Eobang-dong, Gimhae-si, Gyeongsangnam-do, Korea | 82-55-325-1331 |
| 6 | Jeollanam-do | Manna Inc. | 6, Seotae-ri, Hwasun-eup, Hwasun-gun, Jeollanam-do, Korea | 82-61-373-6144 |
| 7 | Jeollabuk-do | Gimje Meat Processing Factory | 630, Guseong-gil, Geumsan-myeon, Gimje-si, Jeollabuk-do, Korea | 82-63-540-6700 |
| 8 | Chungcheongnam-do | Hongju Meat Inc. | 539, Sangjeong-ri, Gwangcheon-eup, Hongseong-gun, Chungcheongnam-do, Korea | 82-41-630-7000 |
| 9 | Chungcheonbuk-do | Farm Story LPC Inc. | 421-3, Seongjae-ri, Ohchang-eup, Choengwon-gun, Chungcheonbuk-do, Korea | 82-43-210-4269 |
| 10 | Gyeongsangbuk-do | Lotte Food Inc. | 94, Gongdan 3-gil, Gimcheon-si, Gyeongsangbuk-do, Korea | 82-54-420-2533 |
| 11 | Jeju-do | Jeju Livestock Joint Market | 2533, Eoeum-ri, Aewol-eup, Jeju-si, Jeju-do, Korea | 82-64-799-5135 |
