# Supplementary material for: Application of LogitBoost Classifier for Traceability Using SNP Chip Data
Source: PLoS One. 2015 Oct 5;10(10):e0139685. doi: 10.1371/journal.pone.0139685 (PMC4593556; doi:10.1371/journal.pone.0139685)
Supplement: S2 Table — Dashes indicate missing information. (DOCX) [file pone.0139685.s005.docx]

**S2 Table**. Selected SNP markers

| **SNP** | **Assay ID** | **SNP name** | **Allele** | **rs number** | **Chromosome** | **Position** |
| --- | --- | --- | --- | --- | --- | --- |
| SNP1 | GTA0029134 | ALGA0002500 | TC | rs81353459 | 1 | 36691125 |
| SNP2 | GTA0029124 | ALGA0003632 | AG | rs80818014 | 1 | 62989866 |
| SNP3 | GTA0029127 | ALGA0005188 | TC | rs81001361 | 1 | 108032798 |
| SNP4 | GTA0027167 | ALGA0010607 | AG | rs81001439 | 1 | 302880686 |
| SNP5 | GTA0027175 | ALGA0012333 | AG | rs81368483 | 2 | 19693385 |
| SNP6 | GTA0027197 | ALGA0017166 | AG | rs81373103 | 3 | 3059882 |
| SNP7 | GTA0027150 | ALGA0017261 | AG | rs81379588 | 3 | 5840882 |
| SNP8 | GTA0029128 | ALGA0020170 | TG | rs81373795 | 3 | 100347076 |
| SNP9 | GTA0027187 | ALGA0020295 | AG | rs81374145 | 3 | 103487445 |
| SNP10 | GTA0027148 | ALGA0023180 | AC | rs80976115 | 4 | 11956752 |
| SNP11 | GTA0027256 | ALGA0026994 | AG | rs81382424 | 4 | 104284028 |
| SNP12 | GTA0029118 | ALGA0028052 | AG | rs81380202 | 4 | 120752641 |
| SNP13 | GTA0027206 | ALGA0030335 | AG | rs81385823 | 5 | 8872938 |
| SNP14 | GTA0029115 | ALGA0033986 | AG | rs80922042 | 5 | 105182709 |
| SNP15 | GTA0027188 | ALGA0034886 | AG | rs81394644 | 6 | 22138877 |
| SNP16 | GTA0029136 | ALGA0037105 | TC | rs81392460 | 6 | 134307926 |
| SNP17 | GTA0027174 | ALGA0038431 | AG | rs80814806 | 7 | 8844382 |
| SNP18 | GTA0027215 | ALGA0038635 | AC | rs80903447 | 7 | 11867962 |
| SNP19 | GTA0029121 | ALGA0043483 | TC | rs80875831 | 7 | 95283610 |
| SNP20 | GTA0027229 | ALGA0052166 | AG | rs81408300 | 9 | 30555946 |
| SNP21 | GTA0027236 | ALGA0056803 | AG | rs81428674 | 10 | 9683745 |
| SNP22 | GTA0029112 | ALGA0056924 | AG | rs81428973 | 10 | 11062697 |
| SNP23 | GTA0027153 | ALGA0059061 | AG | rs81425082 | 10 | 52086866 |
| SNP24 | GTA0029116 | ALGA0064392 | TG | rs81433418 | 12 | 4992763 |
| SNP25 | GTA0029138 | ALGA0065426 | TC | rs81440978 | 12 | 17788471 |
| SNP26 | GTA0029119 | ALGA0067483 | AG | rs80931112 | 13 | 3445254 |
| SNP27 | GTA0027183 | ALGA0071504 | AG | rs81447525 | 13 | 99980492 |
| SNP28 | GTA0029129 | ALGA0072858 | AG | rs80939920 | 13 | 183883486 |
| SNP29 | GTA0027151 | ALGA0073188 | AC | rs81441710 | 13 | 192229132 |
| SNP30 | GTA0027205 | ALGA0075911 | AG | rs80803891 | 14 | 20105162 |
| SNP31 | GTA0029131 | ALGA0079359 | TC | rs80973431 | 14 | 90459793 |
| SNP32 | GTA0027224 | ALGA0083823 | AG | rs80810051 | 15 | 1047733 |
| SNP33 | GTA0027178 | ALGA0084361 | AC | rs81451849 | 15 | 24623255 |
| SNP34 | GTA0029117 | ALGA0085130 | AG | rs80966936 | 15 | 50746568 |
| SNP35 | GTA0027204 | ALGA0088449 | AG | rs81244935 | 15 | 157102798 |
| SNP36 | GTA0029120 | ALGA0089251 | TG | rs81464737 | 16 | 15711247 |
| SNP37 | GTA0029126 | ALGA0092844 | TC | rs80962528 | 17 | 5506024 |
| SNP38 | GTA0027225 | ALGA0093942 | AG | rs81465558 | 17 | 28475777 |
| SNP39 | GTA0027254 | ALGA0095059 | AG | rs80831567 | 17 | 45969331 |
| SNP40 | GTA0029137 | ALGA0097474 | TC | rs81467738 | 18 | 24947919 |
| SNP41 | GTA0029130 | ALGA0097857 | AG | rs81468642 | 18 | 35731229 |
| SNP42 | GTA0027237 | ALGA0109641 | AG | rs81477834 | 11 | 29405328 |
| SNP43 | GTA0027257 | ALGA0110410 | AG | rs81338661 | 2 | 26730378 |
| SNP44 | GTA0027180 | ALGA0115847 | AC | rs81345194 | 8 | 138063274 |
| SNP45 | GTA0027250 | ALGA0119982 | AG | rs81327268 | 13 | 13928440 |
| SNP46 | GTA0029135 | ALGA0124374 | AG | rs81305532 | 3 | 11691606 |
| SNP47 | GTA0027217 | ASGA0001168 | AC | rs81348505 | 1 | 15731768 |
| SNP48 | GTA0027246 | ASGA0003689 | AG | rs81354990 | 1 | 93179162 |
| SNP49 | GTA0027209 | ASGA0006871 | AG | rs81351913 | 1 | 285219286 |
| SNP50 | GTA0027239 | ASGA0009403 | AG | rs81368238 | 2 | 18805832 |
| SNP51 | GTA0027231 | ASGA0011793 | AG | rs81364493 | 2 | 133593694 |
| SNP52 | GTA0027149 | ASGA0017082 | AG | rs80886731 | 4 | 2363714 |
| SNP53 | GTA0027171 | ASGA0018449 | AG | rs80897680 | 4 | 13602527 |
| SNP54 | GTA0027198 | ASGA0029755 | AG | rs80983079 | - | - |
| SNP55 | GTA0027211 | ASGA0031089 | AG | rs80801891 | 7 | 9721700 |
| SNP56 | GTA0027159 | ASGA0035039 | AG | rs80791412 | 7 | 92270821 |
| SNP57 | GTA0027169 | ASGA0035601 | AG | rs81396105 | 7 | 105616538 |
| SNP58 | GTA0029114 | ASGA0040082 | TC | rs81404763 | 8 | 139708737 |
| SNP59 | GTA0027258 | ASGA0041336 | AG | rs81413894 | 9 | 9224376 |
| SNP60 | GTA0027249 | ASGA0042099 | CG | rs81407644 | 9 | 26191661 |
| SNP61 | GTA0027199 | ASGA0048625 | AC | rs81426512 | 10 | 64825425 |
| SNP62 | GTA0027192 | ASGA0060257 | AG | rs81443163 | 13 | 215340754 |
| SNP63 | GTA0027170 | ASGA0060872 | AG | rs81450975 | 14 | 7471763 |
| SNP64 | GTA0027160 | ASGA0094977 | AG | rs81314288 | 3 | 16913373 |
| SNP65 | GTA0029113 | ASGA0096881 | TC | rs81316705 | - | - |
| SNP66 | GTA0029125 | H3GA0000077 | AC | rs81355602 | 1 | 1614750 |
| SNP67 | GTA0027185 | H3GA0000926 | AG | rs80963451 | 1 | 17622619 |
| SNP68 | GTA0027163 | H3GA0006218 | AG | rs81368467 | 6 | 147237891 |
| SNP69 | GTA0027244 | H3GA0009291 | AG | rs81369179 | 3 | 36270941 |
| SNP70 | GTA0027193 | H3GA0012015 | AG | rs80918208 | 4 | 13008743 |
| SNP71 | GTA0027165 | H3GA0026867 | AC | rs81408241 | 9 | 30203501 |
| SNP72 | GTA0027219 | H3GA0027004 | AG | rs81409265 | 9 | 43779851 |
| SNP73 | GTA0029133 | H3GA0028278 | AG | rs81416948 | 9 | 136602162 |
| SNP74 | GTA0027194 | H3GA0030549 | AC | rs81426586 | 10 | 64940895 |
| SNP75 | GTA0029123 | H3GA0031292 | AG | rs80962437 | 11 | 10198249 |
| SNP76 | GTA0027156 | H3GA0031439 | AG | rs81430022 | 11 | 16289120 |
| SNP77 | GTA0027243 | H3GA0038523 | AG | rs80883745 | 14 | 5972386 |
| SNP78 | GTA0027161 | H3GA0046698 | AG | rs81460044 | 16 | 57921341 |
| SNP79 | GTA0027235 | H3GA0048952 | AC | rs80976267 | 17 | 45602515 |
| SNP80 | GTA0027208 | H3GA0054041 | AG | rs81325463 | 1 | 287683176 |
| SNP81 | GTA0027162 | H3GA0056419 | AG | rs81323954 | 12 | 7759970 |
| SNP82 | GTA0029132 | M1GA0001903 | AC | rs80820154 | 1 | 305774749 |
| SNP83 | GTA0027173 | M1GA0008026 | AG | rs81385563 | 5 | 79039700 |
| SNP84 | GTA0027201 | M1GA0011894 | AG | rs81399380 | 8 | 33090664 |
| SNP85 | GTA0027222 | M1GA0018145 | AG | rs80914882 | 14 | 7363912 |
| SNP86 | GTA0027184 | M1GA0022894 | AG | rs80960907 | 17 | 69302804 |
| SNP87 | GTA0027196 | M1GA0024249 | AG | rs81331703 | 12 | 22259328 |
| SNP88 | GTA0027200 | MARC0001787 | AG | - | - | - |
| SNP89 | GTA0029122 | MARC0004720 | TC | - | - | - |
| SNP90 | GTA0027176 | MARC0008528 | AG | - | - | - |
| SNP91 | GTA0027212 | MARC0034477 | AC | - | - | - |
| SNP92 | GTA0027207 | MARC0055696 | AG | - | - | - |
| SNP93 | GTA0027238 | MARC0056053 | AG | - | - | - |
| SNP94 | GTA0027203 | MARC0065987 | AG | - | - | - |
| SNP95 | GTA0027168 | MARC0073259 | AC | - | - | - |
| SNP96 | GTA0027251 | MARC0076283 | AG | - | - | - |

Dashes indicate missing information.
