# Supplementary material for: Application of LogitBoost Classifier for Traceability Using SNP Chip Data
Source: PLoS One. 2015 Oct 5;10(10):e0139685. doi: 10.1371/journal.pone.0139685 (PMC4593556; doi:10.1371/journal.pone.0139685)
Supplement: S3 Table — (DOCX) [file pone.0139685.s006.docx]

**S3 Table**. Feature scores for each SNP calculated with three classifiers and two approaches

**(A)** Kinship ≥ 0.00

|  | **LogitBoost** | | **KNN (IBk)** | | **SVM (SMO)** | |
| --- | --- | --- | --- | --- | --- | --- |
| **SNP** | **Approach 1** | **Approach 2** | **Approach 1** | **Approach 2** | **Approach 1** | **Approach 2** |
| ALGA0003632 | 44.804 | 65.047 | 52.227 | 54.386 | 29.015 | 38.057 |
| ALGA0005188 | 40.756 | 51.417 | 53.981 | 43.725 | 55.870 | 40.081 |
| ALGA0010607 | 34.143 | 46.154 | 24.291 | 25.506 | 32.659 | 41.296 |
| ALGA0012333 | 57.625 | 56.680 | 31.579 | 46.154 | 53.576 | 42.240 |
| ALGA0028052 | 57.895 | 34.143 | 25.506 | 25.776 | 45.884 | 43.860 |
| ALGA0033986 | 61.943 | 53.171 | 11.876 | 36.032 | 41.430 | 46.019 |
| ALGA0034886 | 49.663 | 51.822 | 54.386 | 31.984 | 35.628 | 45.209 |
| ALGA0038635 | 48.313 | 41.296 | 41.700 | 34.413 | 12.686 | 45.614 |
| ALGA0043483 | 40.891 | 56.005 | 53.441 | 46.694 | 56.545 | 46.559 |
| ALGA0056803 | 58.165 | 63.428 | 40.081 | 53.711 | 37.247 | 9.717 |
| ALGA0059061 | 50.607 | 42.915 | 36.707 | 37.787 | 58.974 | 60.999 |
| ALGA0064392 | 44.534 | 55.466 | 12.551 | 46.424 | 10.661 | 51.687 |
| ALGA0067483 | 27.126 | 21.457 | 42.105 | 47.368 | 21.997 | 59.109 |
| ALGA0072858 | 47.368 | 36.842 | 42.375 | 44.669 | 38.596 | 52.632 |
| ALGA0075911 | 58.974 | 16.329 | 46.559 | 15.789 | 8.907 | 8.772 |
| ALGA0085130 | 14.305 | 55.466 | 33.468 | 52.632 | 50.067 | 52.227 |
| ALGA0089251 | 64.642 | 52.632 | 27.126 | 47.638 | 49.798 | 53.036 |
| ALGA0092844 | 61.673 | 45.074 | 20.378 | 29.825 | 22.402 | 52.767 |
| ALGA0093942 | 25.506 | 55.061 | 27.530 | 48.853 | 13.630 | 60.594 |
| ALGA0095059 | 39.676 | 41.296 | 40.621 | 36.032 | 59.649 | 56.410 |
| ALGA0097857 | 41.970 | 46.964 | 29.825 | 31.309 | 38.327 | 56.815 |
| ALGA0110410 | 40.756 | 41.700 | 54.386 | 26.451 | 32.794 | 40.486 |
| ALGA0115847 | 46.829 | 55.331 | 51.147 | 50.202 | 53.441 | 47.638 |
| ALGA0119982 | 58.435 | 28.880 | 14.170 | 20.243 | 10.121 | 59.514 |
| ASGA0006871 | 45.344 | 51.012 | 19.568 | 36.707 | 59.379 | 41.026 |
| ASGA0009403 | 45.344 | 50.607 | 31.714 | 42.375 | 14.575 | 59.244 |
| ASGA0011793 | 36.707 | 48.853 | 50.067 | 38.057 | 44.534 | 43.725 |
| ASGA0017082 | 38.192 | 49.933 | 31.309 | 46.019 | 18.758 | 42.240 |
| ASGA0018449 | 50.877 | 55.466 | 53.441 | 47.503 | 12.281 | 44.804 |
| ASGA0031089 | 39.541 | 54.926 | 41.296 | 49.258 | 51.957 | 46.694 |
| ASGA0035601 | 64.103 | 51.822 | 50.877 | 40.081 | 12.281 | 47.773 |
| ASGA0040082 | 44.939 | 54.386 | 42.915 | 50.472 | 54.386 | 48.853 |
| ASGA0041336 | 63.833 | 59.109 | 50.067 | 49.798 | 39.001 | 48.313 |
| ASGA0042099 | 60.594 | 62.078 | 40.081 | 54.386 | 56.140 | 49.393 |
| ASGA0060257 | 50.067 | 64.777 | 45.074 | 55.331 | 39.001 | 53.576 |
| ASGA0060872 | 47.503 | 51.417 | 53.846 | 47.233 | 46.424 | 59.649 |
| ASGA0094977 | 44.130 | 49.393 | 54.116 | 42.375 | 9.312 | 44.265 |
| ASGA0096881 | 27.395 | 48.853 | 29.555 | 47.503 | 9.717 | 9.042 |
| GTA0027154 | 30.769 | 33.468 | 36.032 | 19.298 | 49.798 | 26.586 |
| GTA0027157 | 34.548 | 27.800 | 41.430 | 13.900 | 56.545 | 32.389 |
| GTA0027158 | 18.084 | 62.753 | 36.302 | 54.386 | 9.312 | 31.174 |
| GTA0027179 | 17.274 | 53.036 | 54.926 | 47.773 | 24.022 | 15.115 |
| GTA0027181 | 11.606 | 65.317 | 51.687 | 54.116 | 40.081 | 15.520 |
| GTA0027182 | 31.849 | 39.946 | 33.738 | 44.265 | 58.030 | 24.561 |
| GTA0027186 | 64.777 | 55.601 | 37.652 | 48.718 | 52.092 | 60.864 |
| GTA0027189 | 36.167 | 48.583 | 21.862 | 37.517 | 27.530 | 34.143 |
| GTA0027213 | 27.260 | 53.846 | 54.521 | 44.130 | 35.897 | 17.139 |
| GTA0027214 | 39.946 | 59.379 | 29.555 | 50.877 | 55.061 | 32.389 |
| GTA0027218 | 51.687 | 46.289 | 40.891 | 42.240 | 12.281 | 21.592 |
| GTA0027230 | 62.078 | 42.240 | 42.240 | 18.489 | 51.012 | 13.765 |
| GTA0027233 | 64.777 | 62.753 | 49.123 | 53.171 | 48.043 | 20.918 |
| GTA0027234 | 64.103 | 28.205 | 29.420 | 35.358 | 48.313 | 8.907 |
| GTA0027240 | 64.372 | 30.769 | 41.430 | 18.084 | 51.417 | 58.300 |
| GTA0027245 | 41.700 | 63.293 | 19.433 | 52.901 | 50.337 | 59.109 |
| GTA0027247 | 33.738 | 46.289 | 53.306 | 26.586 | 40.891 | 24.831 |
| GTA0058588 | 38.866 | 51.687 | 33.738 | 45.074 | 47.638 | 23.887 |
| GTA0058589 | 63.563 | 56.275 | 52.497 | 49.258 | 51.012 | 34.143 |
| GTA0058592 | 45.749 | 48.448 | 33.198 | 39.271 | 32.254 | 58.974 |
| GTA0058594 | 59.379 | 39.136 | 51.417 | 35.088 | 24.966 | 24.022 |
| GTA0058596 | 47.638 | 48.448 | 48.313 | 43.860 | 39.676 | 15.924 |
| GTA0058597 | 48.043 | 52.632 | 36.842 | 48.313 | 53.846 | 30.904 |
| GTA0058598 | 44.130 | 56.005 | 54.386 | 52.767 | 41.161 | 28.205 |
| GTA0058600 | 26.586 | 48.448 | 54.926 | 45.614 | 17.409 | 29.285 |
| GTA0058601 | 7.962 | 22.942 | 23.347 | 45.884 | 8.907 | 34.548 |
| GTA0058602 | 41.565 | 49.258 | 37.112 | 45.479 | 27.530 | 25.236 |
| GTA0059294 | 42.105 | 20.918 | 10.121 | 14.035 | 8.907 | 8.772 |
| GTA0059295 | 24.561 | 58.974 | 54.656 | 51.822 | 46.289 | 55.061 |
| GTA0059296 | 42.915 | 55.196 | 24.291 | 50.877 | 56.545 | 42.915 |
| GTA0059297 | 38.731 | 27.260 | 46.289 | 16.194 | 50.067 | 9.852 |
| GTA0059299 | 63.563 | 63.968 | 20.378 | 55.061 | 11.606 | 52.092 |
| GTA0059301 | 32.389 | 47.638 | 23.617 | 48.178 | 59.109 | 43.320 |
| GTA0059302 | 47.233 | 42.645 | 53.441 | 45.884 | 58.300 | 43.455 |
| GTA0059303 | 28.880 | 50.337 | 50.607 | 45.074 | 9.042 | 50.067 |
| GTA0059304 | 47.773 | 64.777 | 13.090 | 47.773 | 36.437 | 46.289 |
| GTA0059305 | 38.596 | 51.282 | 36.437 | 49.798 | 53.981 | 42.915 |
| GTA0059306 | 39.271 | 22.267 | 31.309 | 16.194 | 45.074 | 41.565 |
| GTA0059307 | 64.103 | 60.459 | 15.655 | 52.767 | 17.409 | 55.870 |
| GTA0059822 | 49.663 | 54.791 | 53.711 | 52.901 | 43.455 | 37.787 |
| H3GA0000077 | 46.964 | 63.023 | 48.718 | 50.607 | 52.497 | 37.922 |
| H3GA0009291 | 45.209 | 42.375 | 24.561 | 32.659 | 49.798 | 9.852 |
| H3GA0012015 | 58.300 | 54.791 | 55.331 | 43.860 | 9.447 | 58.300 |
| H3GA0027004 | 63.833 | 56.275 | 39.001 | 54.116 | 48.448 | 48.853 |
| H3GA0031439 | 27.935 | 48.043 | 54.251 | 34.278 | 54.251 | 50.067 |
| H3GA0046698 | 42.780 | 21.457 | 42.915 | 16.734 | 27.800 | 8.772 |
| H3GA0048952 | 35.223 | 33.063 | 41.970 | 35.628 | 29.960 | 55.601 |
| M1GA0008026 | 7.962 | 7.287 | 42.780 | 6.208 | 21.188 | 8.907 |
| M1GA0011894 | 32.794 | 51.822 | 26.721 | 53.171 | 29.825 | 47.099 |
| MARC0004720 | 31.309 | 48.178 | 27.935 | 43.995 | 55.601 | 10.931 |
| MARC0008528 | 44.669 | 49.798 | 36.437 | 38.731 | 29.960 | 11.606 |
| MARC0055696 | 43.185 | 45.074 | 49.528 | 40.081 | 57.760 | 13.090 |
| MARC0065987 | 60.594 | 40.081 | 47.773 | 31.174 | 59.109 | 58.570 |
| MARC0076283 | 49.528 | 53.441 | 50.067 | 40.891 | 49.393 | 12.955 |
| Mean | 43.951 | 47.775 | 38.944 | 40.889 | 37.652 | 38.205 |
| Variance | 193.173 | 150.206 | 160.087 | 141.233 | 283.874 | 275.354 |

**(B)** Kinship ≥ 0.05

|  | **LogitBoost** | | **KNN (IBk)** | | **SVM (SMO)** | |
| --- | --- | --- | --- | --- | --- | --- |
| **SNP** | **Approach 1** | **Approach 2** | **Approach 1** | **Approach 2** | **Approach 1** | **Approach 2** |
| ALGA0003632 | 78.723 | 47.234 | 46.383 | 39.149 | 69.362 | 52.340 |
| ALGA0005188 | 82.128 | 65.532 | 54.894 | 40.851 | 65.106 | 71.915 |
| ALGA0010607 | 71.489 | 82.553 | 56.596 | 67.660 | 73.617 | 71.489 |
| ALGA0012333 | 81.702 | 78.723 | 58.298 | 68.511 | 71.915 | 25.532 |
| ALGA0028052 | 82.979 | 83.830 | 66.383 | 69.787 | 75.745 | 76.596 |
| ALGA0033986 | 72.340 | 65.106 | 55.745 | 57.021 | 67.234 | 71.064 |
| ALGA0034886 | 80.000 | 47.660 | 57.447 | 42.979 | 42.553 | 74.894 |
| ALGA0038635 | 79.574 | 85.106 | 54.894 | 68.085 | 68.511 | 71.064 |
| ALGA0043483 | 82.128 | 82.553 | 22.979 | 66.809 | 72.340 | 72.340 |
| ALGA0056803 | 77.872 | 74.468 | 38.723 | 62.128 | 72.766 | 75.745 |
| ALGA0059061 | 81.702 | 51.489 | 57.872 | 43.830 | 67.660 | 77.447 |
| ALGA0064392 | 77.872 | 82.128 | 67.660 | 71.064 | 65.532 | 53.617 |
| ALGA0067483 | 21.702 | 31.064 | 44.681 | 65.957 | 32.766 | 18.298 |
| ALGA0072858 | 83.830 | 73.617 | 54.894 | 67.234 | 33.617 | 77.021 |
| ALGA0075911 | 83.404 | 63.830 | 67.660 | 61.702 | 62.979 | 78.298 |
| ALGA0085130 | 30.638 | 81.277 | 60.851 | 66.383 | 17.872 | 80.000 |
| ALGA0089251 | 75.319 | 82.128 | 51.489 | 67.660 | 74.894 | 77.021 |
| ALGA0092844 | 65.957 | 72.340 | 57.447 | 60.851 | 72.340 | 79.574 |
| ALGA0093942 | 82.979 | 73.617 | 48.085 | 62.979 | 73.191 | 77.872 |
| ALGA0095059 | 82.553 | 82.979 | 41.277 | 63.404 | 35.319 | 77.872 |
| ALGA0097857 | 83.830 | 85.532 | 57.872 | 64.681 | 71.064 | 79.574 |
| ALGA0110410 | 68.085 | 82.128 | 35.745 | 64.681 | 18.723 | 54.043 |
| ALGA0115847 | 75.319 | 67.660 | 65.532 | 66.809 | 61.702 | 74.043 |
| ALGA0119982 | 79.149 | 82.128 | 67.660 | 68.511 | 22.128 | 77.021 |
| ASGA0006871 | 64.255 | 75.319 | 60.851 | 64.681 | 51.915 | 27.660 |
| ASGA0009403 | 82.553 | 82.553 | 37.447 | 65.957 | 42.128 | 76.596 |
| ASGA0011793 | 83.404 | 64.681 | 51.915 | 63.830 | 74.468 | 35.319 |
| ASGA0017082 | 74.043 | 77.021 | 63.830 | 64.255 | 52.766 | 77.021 |
| ASGA0018449 | 72.766 | 78.298 | 66.383 | 67.660 | 54.043 | 56.596 |
| ASGA0031089 | 69.787 | 74.043 | 69.362 | 43.830 | 70.638 | 71.915 |
| ASGA0035601 | 69.362 | 70.638 | 66.383 | 54.894 | 68.085 | 57.447 |
| ASGA0040082 | 82.128 | 83.404 | 45.957 | 63.404 | 74.468 | 28.085 |
| ASGA0041336 | 80.851 | 77.021 | 45.957 | 62.979 | 71.915 | 36.170 |
| ASGA0042099 | 47.234 | 80.000 | 59.149 | 58.298 | 65.106 | 76.596 |
| ASGA0060257 | 72.340 | 82.128 | 68.085 | 67.234 | 42.553 | 17.872 |
| ASGA0060872 | 71.489 | 74.043 | 57.021 | 66.383 | 74.043 | 78.723 |
| ASGA0094977 | 69.787 | 84.681 | 68.085 | 63.830 | 61.277 | 73.617 |
| ASGA0096881 | 72.340 | 69.787 | 60.426 | 68.511 | 74.043 | 78.298 |
| GTA0027154 | 64.681 | 82.128 | 61.277 | 66.809 | 73.191 | 70.213 |
| GTA0027157 | 81.277 | 69.787 | 60.851 | 64.255 | 67.234 | 70.638 |
| GTA0027158 | 80.000 | 83.830 | 43.404 | 68.085 | 25.532 | 73.191 |
| GTA0027179 | 80.426 | 70.213 | 57.447 | 55.745 | 21.702 | 61.702 |
| GTA0027181 | 65.957 | 54.043 | 52.766 | 35.319 | 74.043 | 17.447 |
| GTA0027182 | 71.064 | 74.468 | 57.447 | 63.404 | 60.000 | 24.681 |
| GTA0027186 | 71.064 | 63.404 | 33.617 | 51.915 | 60.000 | 55.319 |
| GTA0027189 | 80.851 | 66.809 | 53.191 | 65.106 | 62.128 | 75.319 |
| GTA0027213 | 77.021 | 82.553 | 44.681 | 67.234 | 37.021 | 48.936 |
| GTA0027214 | 81.702 | 84.255 | 41.277 | 63.404 | 54.043 | 77.872 |
| GTA0027218 | 65.957 | 47.660 | 40.426 | 39.149 | 24.681 | 19.149 |
| GTA0027230 | 77.447 | 82.553 | 17.872 | 65.532 | 50.213 | 78.298 |
| GTA0027233 | 82.128 | 71.915 | 37.447 | 52.766 | 53.191 | 62.553 |
| GTA0027234 | 84.681 | 70.638 | 57.872 | 65.532 | 69.362 | 30.638 |
| GTA0027240 | 70.638 | 68.085 | 51.064 | 64.255 | 72.766 | 63.404 |
| GTA0027245 | 37.021 | 56.170 | 57.021 | 43.830 | 41.702 | 76.170 |
| GTA0027247 | 78.298 | 71.064 | 50.638 | 45.957 | 53.617 | 29.362 |
| GTA0058588 | 82.979 | 75.319 | 41.702 | 67.660 | 53.617 | 65.106 |
| GTA0058589 | 75.745 | 78.298 | 48.511 | 62.979 | 28.085 | 76.596 |
| GTA0058592 | 80.851 | 76.596 | 59.149 | 66.809 | 75.319 | 22.553 |
| GTA0058594 | 80.000 | 73.191 | 51.915 | 64.255 | 68.085 | 62.553 |
| GTA0058596 | 83.404 | 52.340 | 59.574 | 40.426 | 51.064 | 65.106 |
| GTA0058597 | 78.298 | 86.383 | 56.170 | 68.511 | 42.553 | 70.213 |
| GTA0058598 | 69.787 | 56.596 | 51.489 | 55.745 | 66.809 | 76.596 |
| GTA0058600 | 67.660 | 67.234 | 56.170 | 56.596 | 73.191 | 70.213 |
| GTA0058601 | 28.936 | 17.872 | 56.596 | 39.574 | 20.851 | 71.489 |
| GTA0058602 | 80.426 | 63.830 | 64.255 | 51.064 | 17.872 | 56.170 |
| GTA0059294 | 70.213 | 80.000 | 51.489 | 65.106 | 68.085 | 77.872 |
| GTA0059295 | 60.000 | 80.000 | 68.936 | 63.404 | 46.809 | 60.000 |
| GTA0059296 | 77.021 | 80.000 | 57.447 | 63.830 | 64.255 | 35.319 |
| GTA0059297 | 78.298 | 77.021 | 66.809 | 64.255 | 73.617 | 74.468 |
| GTA0059299 | 80.000 | 70.213 | 60.426 | 59.149 | 72.766 | 39.574 |
| GTA0059301 | 65.532 | 84.681 | 64.681 | 67.234 | 76.596 | 32.340 |
| GTA0059302 | 82.979 | 66.809 | 54.043 | 52.340 | 67.234 | 72.340 |
| GTA0059303 | 82.128 | 63.830 | 47.234 | 56.596 | 41.702 | 20.426 |
| GTA0059304 | 72.340 | 72.766 | 65.532 | 56.170 | 57.447 | 16.170 |
| GTA0059305 | 71.064 | 68.511 | 59.149 | 55.745 | 62.553 | 75.745 |
| GTA0059306 | 81.277 | 74.468 | 65.957 | 60.000 | 66.383 | 73.191 |
| GTA0059307 | 72.340 | 78.298 | 65.106 | 66.809 | 41.277 | 41.277 |
| GTA0059822 | 67.234 | 68.511 | 29.362 | 55.319 | 72.766 | 55.319 |
| H3GA0000077 | 84.681 | 33.617 | 57.872 | 25.106 | 30.213 | 74.468 |
| H3GA0009291 | 82.553 | 77.021 | 58.298 | 66.809 | 69.787 | 73.617 |
| H3GA0012015 | 72.766 | 72.766 | 54.894 | 62.979 | 74.894 | 73.191 |
| H3GA0027004 | 73.191 | 71.064 | 66.809 | 57.447 | 54.894 | 74.894 |
| H3GA0031439 | 80.000 | 84.681 | 54.043 | 67.660 | 41.702 | 77.872 |
| H3GA0046698 | 77.021 | 72.766 | 51.915 | 63.404 | 69.787 | 78.298 |
| H3GA0048952 | 77.872 | 75.319 | 66.809 | 58.298 | 61.277 | 42.979 |
| M1GA0008026 | 26.383 | 20.851 | 53.617 | 19.574 | 68.085 | 35.745 |
| M1GA0011894 | 77.447 | 43.830 | 35.745 | 28.936 | 52.766 | 74.468 |
| MARC0004720 | 77.021 | 74.468 | 68.936 | 63.404 | 66.383 | 63.404 |
| MARC0008528 | 79.574 | 80.000 | 40.851 | 65.532 | 44.681 | 77.021 |
| MARC0055696 | 82.979 | 68.085 | 58.723 | 64.681 | 68.511 | 65.957 |
| MARC0065987 | 85.106 | 39.149 | 24.681 | 33.191 | 51.915 | 45.532 |
| MARC0076283 | 80.426 | 49.787 | 67.234 | 42.128 | 74.468 | 45.957 |
| Mean | 73.797 | 70.370 | 54.265 | 58.821 | 57.664 | 60.712 |
| Variance | 161.363 | 203.136 | 124.054 | 122.254 | 283.227 | 389.687 |

**(C)** Kinship ≥ 0.10

|  | **LogitBoost** | | **KNN (IBk)** | | **SVM (SMO)** | |
| --- | --- | --- | --- | --- | --- | --- |
| **SNP** | **Approach 1** | **Approach 2** | **Approach 1** | **Approach 2** | **Approach 1** | **Approach 2** |
| ALGA0003632 | 44.776 | 91.045 | 54.478 | 75.373 | 73.881 | 70.149 |
| ALGA0005188 | 82.090 | 64.925 | 32.836 | 41.791 | 54.478 | 29.851 |
| ALGA0010607 | 82.836 | 90.299 | 64.925 | 76.119 | 76.866 | 73.881 |
| ALGA0012333 | 65.672 | 93.284 | 74.627 | 74.627 | 69.403 | 73.134 |
| ALGA0028052 | 81.343 | 88.060 | 73.134 | 72.388 | 71.642 | 76.866 |
| ALGA0033986 | 83.582 | 88.806 | 57.463 | 74.627 | 67.910 | 74.627 |
| ALGA0034886 | 93.284 | 91.791 | 75.373 | 78.358 | 73.134 | 76.866 |
| ALGA0038635 | 50.000 | 92.537 | 76.119 | 79.104 | 71.642 | 75.373 |
| ALGA0043483 | 79.851 | 88.806 | 76.119 | 70.149 | 70.896 | 74.627 |
| ALGA0056803 | 92.537 | 90.299 | 70.896 | 74.627 | 68.657 | 70.896 |
| ALGA0059061 | 77.612 | 91.791 | 76.119 | 72.388 | 74.627 | 73.881 |
| ALGA0064392 | 91.045 | 90.299 | 56.716 | 74.627 | 39.552 | 29.851 |
| ALGA0067483 | 56.716 | 38.806 | 38.806 | 42.537 | 76.866 | 41.791 |
| ALGA0072858 | 60.448 | 92.537 | 73.881 | 77.612 | 76.119 | 71.642 |
| ALGA0075911 | 84.328 | 88.806 | 74.627 | 74.627 | 52.239 | 75.373 |
| ALGA0085130 | 93.284 | 92.537 | 78.358 | 77.612 | 29.851 | 73.881 |
| ALGA0089251 | 91.045 | 92.537 | 54.478 | 74.627 | 72.388 | 73.881 |
| ALGA0092844 | 82.836 | 91.791 | 55.970 | 76.119 | 70.896 | 76.119 |
| ALGA0093942 | 80.597 | 88.806 | 73.134 | 74.627 | 55.970 | 40.299 |
| ALGA0095059 | 63.433 | 89.552 | 68.657 | 77.612 | 72.388 | 73.134 |
| ALGA0097857 | 88.060 | 91.045 | 53.731 | 78.358 | 74.627 | 71.642 |
| ALGA0110410 | 91.791 | 91.045 | 75.373 | 72.388 | 73.881 | 68.657 |
| ALGA0115847 | 91.791 | 65.672 | 70.896 | 58.955 | 50.746 | 71.642 |
| ALGA0119982 | 63.433 | 92.537 | 78.358 | 77.612 | 76.119 | 72.388 |
| ASGA0006871 | 61.194 | 90.299 | 72.388 | 77.612 | 70.149 | 76.866 |
| ASGA0009403 | 81.343 | 93.284 | 73.881 | 75.373 | 70.896 | 73.134 |
| ASGA0011793 | 81.343 | 87.313 | 61.194 | 77.612 | 66.418 | 72.388 |
| ASGA0017082 | 61.940 | 91.791 | 64.179 | 79.851 | 35.075 | 75.373 |
| ASGA0018449 | 80.597 | 90.299 | 51.493 | 53.731 | 72.388 | 71.642 |
| ASGA0031089 | 64.179 | 91.791 | 78.358 | 79.104 | 68.657 | 73.134 |
| ASGA0035601 | 63.433 | 91.791 | 76.866 | 78.358 | 70.149 | 75.373 |
| ASGA0040082 | 64.179 | 91.045 | 74.627 | 77.612 | 34.328 | 77.612 |
| ASGA0041336 | 81.343 | 92.537 | 71.642 | 80.597 | 75.373 | 70.149 |
| ASGA0042099 | 94.776 | 88.060 | 74.627 | 73.881 | 69.403 | 70.896 |
| ASGA0060257 | 92.537 | 92.537 | 70.896 | 73.881 | 68.657 | 72.388 |
| ASGA0060872 | 79.104 | 90.299 | 74.627 | 80.597 | 73.134 | 73.134 |
| ASGA0094977 | 79.104 | 91.791 | 61.940 | 78.358 | 58.209 | 76.119 |
| ASGA0096881 | 82.090 | 86.567 | 70.896 | 76.119 | 75.373 | 47.015 |
| GTA0027154 | 83.582 | 93.284 | 73.134 | 76.119 | 72.388 | 76.866 |
| GTA0027157 | 93.284 | 91.791 | 76.866 | 77.612 | 74.627 | 67.164 |
| GTA0027158 | 92.537 | 91.045 | 72.388 | 61.194 | 50.746 | 75.373 |
| GTA0027179 | 93.284 | 44.030 | 67.910 | 43.284 | 61.194 | 58.209 |
| GTA0027181 | 82.090 | 89.552 | 67.164 | 73.881 | 29.851 | 61.940 |
| GTA0027182 | 81.343 | 90.299 | 77.612 | 76.866 | 75.373 | 65.672 |
| GTA0027186 | 59.701 | 87.313 | 52.239 | 66.418 | 70.896 | 76.119 |
| GTA0027189 | 82.090 | 88.806 | 70.149 | 73.881 | 73.881 | 41.045 |
| GTA0027213 | 83.582 | 91.045 | 73.881 | 77.612 | 76.119 | 61.194 |
| GTA0027214 | 64.925 | 90.299 | 74.627 | 73.881 | 42.537 | 66.418 |
| GTA0027218 | 84.328 | 41.791 | 70.149 | 47.761 | 52.985 | 63.433 |
| GTA0027230 | 73.881 | 91.045 | 53.731 | 79.104 | 66.418 | 59.701 |
| GTA0027233 | 70.149 | 91.791 | 67.910 | 78.358 | 77.612 | 64.179 |
| GTA0027234 | 82.836 | 85.821 | 70.149 | 73.134 | 73.134 | 41.791 |
| GTA0027240 | 79.851 | 93.284 | 67.910 | 79.851 | 61.194 | 71.642 |
| GTA0027245 | 92.537 | 67.910 | 55.970 | 66.418 | 42.537 | 63.433 |
| GTA0027247 | 91.791 | 90.299 | 76.866 | 74.627 | 42.537 | 63.433 |
| GTA0058588 | 82.090 | 93.284 | 76.119 | 77.612 | 29.851 | 76.119 |
| GTA0058589 | 75.373 | 91.791 | 71.642 | 73.881 | 72.388 | 76.119 |
| GTA0058592 | 93.284 | 93.284 | 67.910 | 76.119 | 42.537 | 73.881 |
| GTA0058594 | 89.552 | 91.045 | 78.358 | 74.627 | 72.388 | 73.881 |
| GTA0058596 | 76.866 | 86.567 | 71.642 | 73.881 | 59.701 | 29.851 |
| GTA0058597 | 82.836 | 88.806 | 73.134 | 65.672 | 67.910 | 70.149 |
| GTA0058598 | 76.119 | 87.313 | 72.388 | 75.373 | 49.254 | 71.642 |
| GTA0058600 | 66.418 | 94.776 | 73.134 | 77.612 | 75.373 | 78.358 |
| GTA0058601 | 38.060 | 43.284 | 58.209 | 65.672 | 29.851 | 29.851 |
| GTA0058602 | 83.582 | 88.806 | 71.642 | 73.881 | 32.090 | 69.403 |
| GTA0059294 | 81.343 | 86.567 | 79.851 | 73.881 | 49.254 | 72.388 |
| GTA0059295 | 70.149 | 55.970 | 61.194 | 49.254 | 77.612 | 47.015 |
| GTA0059296 | 43.284 | 87.313 | 71.642 | 78.358 | 52.985 | 70.896 |
| GTA0059297 | 94.030 | 88.060 | 76.866 | 60.448 | 72.388 | 72.388 |
| GTA0059299 | 79.104 | 91.791 | 73.134 | 74.627 | 76.866 | 41.791 |
| GTA0059301 | 45.522 | 91.045 | 74.627 | 79.851 | 80.597 | 38.806 |
| GTA0059302 | 93.284 | 91.791 | 71.642 | 75.373 | 47.015 | 70.149 |
| GTA0059303 | 93.284 | 93.284 | 72.388 | 75.373 | 52.239 | 72.388 |
| GTA0059304 | 92.537 | 92.537 | 73.881 | 77.612 | 73.134 | 74.627 |
| GTA0059305 | 85.075 | 93.284 | 71.642 | 79.851 | 73.881 | 76.866 |
| GTA0059306 | 76.866 | 92.537 | 74.627 | 75.373 | 69.403 | 67.910 |
| GTA0059307 | 92.537 | 92.537 | 78.358 | 75.373 | 72.388 | 76.119 |
| GTA0059822 | 94.030 | 90.299 | 71.642 | 56.716 | 63.433 | 70.149 |
| H3GA0000077 | 85.075 | 52.239 | 67.910 | 43.284 | 62.687 | 71.642 |
| H3GA0009291 | 61.194 | 92.537 | 73.134 | 73.134 | 71.642 | 73.134 |
| H3GA0012015 | 94.776 | 91.791 | 63.433 | 64.179 | 58.955 | 73.134 |
| H3GA0027004 | 92.537 | 89.552 | 64.179 | 76.866 | 70.896 | 41.045 |
| H3GA0031439 | 65.672 | 91.045 | 72.388 | 80.597 | 49.254 | 72.388 |
| H3GA0046698 | 81.343 | 89.552 | 74.627 | 74.627 | 64.925 | 73.134 |
| H3GA0048952 | 85.075 | 89.552 | 77.612 | 76.119 | 72.388 | 76.119 |
| M1GA0008026 | 82.836 | 90.299 | 64.925 | 76.119 | 56.716 | 29.851 |
| M1GA0011894 | 81.343 | 92.537 | 71.642 | 75.373 | 58.209 | 72.388 |
| MARC0004720 | 77.612 | 91.045 | 71.642 | 79.104 | 73.881 | 49.254 |
| MARC0008528 | 65.672 | 90.299 | 49.254 | 76.119 | 59.701 | 47.015 |
| MARC0055696 | 38.806 | 91.791 | 65.672 | 59.701 | 73.881 | 56.716 |
| MARC0065987 | 74.627 | 89.552 | 73.134 | 75.373 | 29.851 | 58.955 |
| MARC0076283 | 76.866 | 88.806 | 70.149 | 76.866 | 61.940 | 76.866 |
| Mean | 78.261 | 86.989 | 69.095 | 72.429 | 63.092 | 65.931 |
| Variance | 188.387 | 143.395 | 77.211 | 83.236 | 195.367 | 181.144 |

**(D)** Kinship ≥ 0.15

|  | **LogitBoost** | | **KNN (IBk)** | | **SVM (SMO)** | |
| --- | --- | --- | --- | --- | --- | --- |
| **SNP** | **Approach 1** | **Approach 2** | **Approach 1** | **Approach 2** | **Approach 1** | **Approach 2** |
| ALGA0003632 | 89.552 | 98.507 | 67.164 | 77.612 | 59.701 | 73.134 |
| ALGA0005188 | 86.567 | 98.507 | 82.090 | 83.582 | 59.701 | 74.627 |
| ALGA0010607 | 82.090 | 97.015 | 70.149 | 88.060 | 85.075 | 77.612 |
| ALGA0012333 | 85.075 | 98.507 | 83.582 | 83.582 | 91.045 | 77.612 |
| ALGA0028052 | 94.030 | 98.507 | 91.045 | 89.552 | 88.060 | 85.075 |
| ALGA0033986 | 89.552 | 98.507 | 91.045 | 94.030 | 89.552 | 89.552 |
| ALGA0034886 | 88.060 | 97.015 | 91.045 | 92.537 | 85.075 | 89.552 |
| ALGA0038635 | 89.552 | 95.522 | 89.552 | 89.552 | 92.537 | 89.552 |
| ALGA0043483 | 89.552 | 97.015 | 91.045 | 91.045 | 59.701 | 89.552 |
| ALGA0056803 | 98.507 | 98.507 | 94.030 | 95.522 | 88.060 | 91.045 |
| ALGA0059061 | 98.507 | 98.507 | 89.552 | 88.060 | 61.194 | 91.045 |
| ALGA0064392 | 97.015 | 97.015 | 89.552 | 89.552 | 91.045 | 91.045 |
| ALGA0067483 | 98.507 | 50.746 | 73.134 | 53.731 | 59.701 | 59.701 |
| ALGA0072858 | 98.507 | 98.507 | 91.045 | 95.522 | 71.642 | 91.045 |
| ALGA0075911 | 98.507 | 98.507 | 95.522 | 91.045 | 86.567 | 59.701 |
| ALGA0085130 | 98.507 | 98.507 | 89.552 | 92.537 | 88.060 | 91.045 |
| ALGA0089251 | 98.507 | 97.015 | 88.060 | 95.522 | 88.060 | 88.060 |
| ALGA0092844 | 98.507 | 97.015 | 91.045 | 82.090 | 80.597 | 59.701 |
| ALGA0093942 | 98.507 | 97.015 | 89.552 | 89.552 | 85.075 | 92.537 |
| ALGA0095059 | 98.507 | 95.522 | 86.567 | 92.537 | 88.060 | 94.030 |
| ALGA0097857 | 98.507 | 98.507 | 88.060 | 94.030 | 59.701 | 92.537 |
| ALGA0110410 | 86.567 | 98.507 | 68.657 | 94.030 | 85.075 | 76.119 |
| ALGA0115847 | 94.030 | 97.015 | 89.552 | 85.075 | 62.687 | 92.537 |
| ALGA0119982 | 98.507 | 98.507 | 91.045 | 95.522 | 85.075 | 92.537 |
| ASGA0006871 | 83.582 | 97.015 | 83.582 | 95.522 | 68.657 | 76.119 |
| ASGA0009403 | 85.075 | 98.507 | 92.537 | 94.030 | 92.537 | 73.134 |
| ASGA0011793 | 91.045 | 98.507 | 91.045 | 91.045 | 86.567 | 91.045 |
| ASGA0017082 | 89.552 | 98.507 | 92.537 | 91.045 | 83.582 | 91.045 |
| ASGA0018449 | 91.045 | 98.507 | 94.030 | 94.030 | 71.642 | 89.552 |
| ASGA0031089 | 89.552 | 98.507 | 91.045 | 92.537 | 89.552 | 89.552 |
| ASGA0035601 | 94.030 | 97.015 | 89.552 | 88.060 | 74.627 | 89.552 |
| ASGA0040082 | 94.030 | 98.507 | 91.045 | 94.030 | 88.060 | 89.552 |
| ASGA0041336 | 95.522 | 98.507 | 92.537 | 91.045 | 88.060 | 89.552 |
| ASGA0042099 | 98.507 | 98.507 | 95.522 | 79.104 | 88.060 | 92.537 |
| ASGA0060257 | 98.507 | 98.507 | 91.045 | 83.582 | 76.119 | 92.537 |
| ASGA0060872 | 98.507 | 97.015 | 89.552 | 85.075 | 89.552 | 91.045 |
| ASGA0094977 | 89.552 | 98.507 | 91.045 | 95.522 | 59.701 | 88.060 |
| ASGA0096881 | 64.179 | 98.507 | 64.179 | 83.582 | 82.090 | 59.701 |
| GTA0027154 | 85.075 | 98.507 | 92.537 | 94.030 | 86.567 | 70.149 |
| GTA0027157 | 88.060 | 98.507 | 91.045 | 80.597 | 59.701 | 70.149 |
| GTA0027158 | 83.582 | 98.507 | 92.537 | 83.582 | 85.075 | 73.134 |
| GTA0027179 | 83.582 | 98.507 | 79.104 | 88.060 | 82.090 | 59.701 |
| GTA0027181 | 86.567 | 98.507 | 74.627 | 85.075 | 59.701 | 59.701 |
| GTA0027182 | 76.119 | 98.507 | 88.060 | 83.582 | 82.090 | 61.194 |
| GTA0027186 | 86.567 | 62.687 | 91.045 | 52.239 | 62.687 | 59.701 |
| GTA0027189 | 95.522 | 98.507 | 92.537 | 85.075 | 82.090 | 68.657 |
| GTA0027213 | 77.612 | 98.507 | 70.149 | 83.582 | 88.060 | 61.194 |
| GTA0027214 | 77.612 | 98.507 | 92.537 | 85.075 | 80.597 | 70.149 |
| GTA0027218 | 80.597 | 98.507 | 77.612 | 83.582 | 59.701 | 62.687 |
| GTA0027230 | 88.060 | 95.522 | 91.045 | 82.090 | 61.194 | 59.701 |
| GTA0027233 | 83.582 | 98.507 | 77.612 | 86.567 | 59.701 | 62.687 |
| GTA0027234 | 77.612 | 97.015 | 91.045 | 82.090 | 59.701 | 73.134 |
| GTA0027240 | 83.582 | 98.507 | 94.030 | 86.567 | 82.090 | 68.657 |
| GTA0027245 | 76.119 | 98.507 | 89.552 | 85.075 | 95.522 | 65.672 |
| GTA0027247 | 80.597 | 98.507 | 89.552 | 83.582 | 85.075 | 62.687 |
| GTA0058588 | 85.075 | 97.015 | 88.060 | 83.582 | 59.701 | 65.672 |
| GTA0058589 | 89.552 | 98.507 | 91.045 | 83.582 | 79.104 | 67.164 |
| GTA0058592 | 82.090 | 98.507 | 79.104 | 79.104 | 85.075 | 59.701 |
| GTA0058594 | 74.627 | 98.507 | 76.119 | 83.582 | 59.701 | 61.194 |
| GTA0058596 | 73.134 | 98.507 | 74.627 | 85.075 | 83.582 | 59.701 |
| GTA0058597 | 83.582 | 98.507 | 59.701 | 86.567 | 82.090 | 68.657 |
| GTA0058598 | 88.060 | 98.507 | 88.060 | 80.597 | 89.552 | 70.149 |
| GTA0058600 | 82.090 | 97.015 | 79.104 | 82.090 | 59.701 | 64.179 |
| GTA0058601 | 85.075 | 98.507 | 70.149 | 86.567 | 79.104 | 68.657 |
| GTA0058602 | 82.090 | 98.507 | 71.642 | 83.582 | 82.090 | 62.687 |
| GTA0059294 | 98.507 | 98.507 | 65.672 | 83.582 | 83.582 | 91.045 |
| GTA0059295 | 98.507 | 46.269 | 91.045 | 46.269 | 76.119 | 94.030 |
| GTA0059296 | 85.075 | 97.015 | 92.537 | 82.090 | 89.552 | 88.060 |
| GTA0059297 | 98.507 | 98.507 | 91.045 | 98.507 | 61.194 | 91.045 |
| GTA0059299 | 98.507 | 98.507 | 92.537 | 91.045 | 89.552 | 89.552 |
| GTA0059301 | 94.030 | 98.507 | 92.537 | 94.030 | 83.582 | 85.075 |
| GTA0059302 | 89.552 | 98.507 | 58.209 | 91.045 | 68.657 | 89.552 |
| GTA0059303 | 98.507 | 98.507 | 94.030 | 76.119 | 86.567 | 89.552 |
| GTA0059304 | 92.537 | 98.507 | 91.045 | 95.522 | 64.179 | 85.075 |
| GTA0059305 | 92.537 | 98.507 | 92.537 | 95.522 | 59.701 | 89.552 |
| GTA0059306 | 88.060 | 98.507 | 92.537 | 94.030 | 64.179 | 77.612 |
| GTA0059307 | 98.507 | 98.507 | 92.537 | 94.030 | 82.090 | 91.045 |
| GTA0059822 | 89.552 | 98.507 | 89.552 | 79.104 | 59.701 | 67.164 |
| H3GA0000077 | 89.552 | 98.507 | 89.552 | 95.522 | 59.701 | 68.657 |
| H3GA0009291 | 94.030 | 98.507 | 92.537 | 88.060 | 89.552 | 91.045 |
| H3GA0012015 | 92.537 | 98.507 | 92.537 | 86.567 | 89.552 | 88.060 |
| H3GA0027004 | 98.507 | 97.015 | 92.537 | 89.552 | 73.134 | 89.552 |
| H3GA0031439 | 98.507 | 98.507 | 88.060 | 83.582 | 86.567 | 89.552 |
| H3GA0046698 | 98.507 | 98.507 | 73.134 | 91.045 | 76.119 | 91.045 |
| H3GA0048952 | 98.507 | 98.507 | 94.030 | 94.030 | 91.045 | 91.045 |
| M1GA0008026 | 94.030 | 98.507 | 68.657 | 89.552 | 88.060 | 89.552 |
| M1GA0011894 | 88.060 | 98.507 | 89.552 | 80.597 | 88.060 | 88.060 |
| MARC0004720 | 70.149 | 98.507 | 86.567 | 83.582 | 80.597 | 59.701 |
| MARC0008528 | 67.164 | 98.507 | 89.552 | 86.567 | 62.687 | 59.701 |
| MARC0055696 | 61.194 | 98.507 | 79.104 | 77.612 | 88.060 | 59.701 |
| MARC0065987 | 82.090 | 98.507 | 82.090 | 91.045 | 94.030 | 59.701 |
| MARC0076283 | 88.060 | 98.507 | 82.090 | 79.104 | 59.701 | 59.701 |
| Mean | 89.082 | 96.674 | 85.967 | 86.567 | 77.790 | 78.066 |
| Variance | 73.779 | 66.443 | 79.611 | 72.558 | 143.787 | 168.017 |
