# Supplementary material for: Application of LogitBoost Classifier for Traceability Using SNP Chip Data
Source: PLoS One. 2015 Oct 5;10(10):e0139685. doi: 10.1371/journal.pone.0139685 (PMC4593556; doi:10.1371/journal.pone.0139685)
Supplement: S4 Table — (DOCX) [file pone.0139685.s007.docx]

**S4 Table.** AUC values for each class calculated with LogitBoost and two approaches

| **Class** | **Approach 1** | **Approach 2** |
| --- | --- | --- |
| **Kinship** ≥ **0.00** | | |
| D1 | 0.902 | 0.848 |
| D2 | 0.970 | 0.952 |
| D10 | 0.866 | 0.811 |
| D11 | 0.930 | 0.907 |
| D13 | 0.934 | 0.944 |
| D18 | 0.945 | 0.941 |
| D27 | 0.868 | 0.848 |
| D38 | 0.942 | 0.929 |
| D59 | 0.984 | 0.744 |
| D60 | 0.989 | 0.857 |
| D61 | 0.798 | 0.738 |
| D62 | 0.987 | 0.988 |
| D66 | 0.872 | 0.836 |
| D89 | 0.635 | 0.906 |
| D90 | 0.973 | 0.914 |
| D100 | 0.976 | 0.976 |
| D102 | 0.976 | 0.930 |
| D103 | 0.846 | 0.821 |
| D107 | 0.968 | 0.927 |
| D114 | 0.943 | 0.927 |
| Mean ± Variance | 0.915 ± 0.007 | 0.887 ± 0.005 |
| **Kinship** ≥**0.05** | | |
| D11 | 0.997 | 0.993 |
| D59 | 0.943 | 0.933 |
| D60 | 0.955 | 0.952 |
| D62 | 1.000 | 1.000 |
| D66 | 0.909 | 0.964 |
| D89 | 0.662 | 0.748 |
| D90 | 0.949 | 0.974 |
| D100 | 0.991 | 0.991 |
| Mean ± Variance | 0.926 ± 0.012 | 0.944 ± 0.007 |
| **Kinship** ≥ **0.10** | | |
| D59 | 0.895 | 1.000 |
| D62 | 0.982 | 1.000 |
| D66 | 0.915 | 0.982 |
| D89 | 0.833 | 1.000 |
| D100 | 0.950 | 0.972 |
| Mean ± Variance | 0.915 ± 0.003 | 0.991 ± 0.000 |
| **Kinship** ≥ **0.15** | | |
| D59 | 0.993 | 1.000 |
| D100 | 0.993 | 1.000 |
| Mean ± Variance | 0.993 ± 0.000 | 1.000 ± 0.000 |
